# Concentrations of Volatile Methyl Siloxanes in New York City Reflect Emissions from Personal Care and Industrial Use

**DOI:** 10.1021/acs.est.3c10752

**Published:** 2024-05-09

**Authors:** Christopher E. Brunet, Rachel F. Marek, Charles O. Stanier, Keri C. Hornbuckle

**Affiliations:** † Department of Civil and Environmental Engineering, IIHR-Hydroscience & Engineering, University of Iowa, Iowa City Iowa 52242, United States; ‡ Department of Chemical and Biochemical Engineering, IIHR-Hydroscience and Engineering, The University of Iowa, Iowa City Iowa 52242, United States

**Keywords:** volatile methyl siloxanes, volatile organic compounds, urban air quality, oxidation, diurnal, New York City, outdoor air, emissions

## Abstract

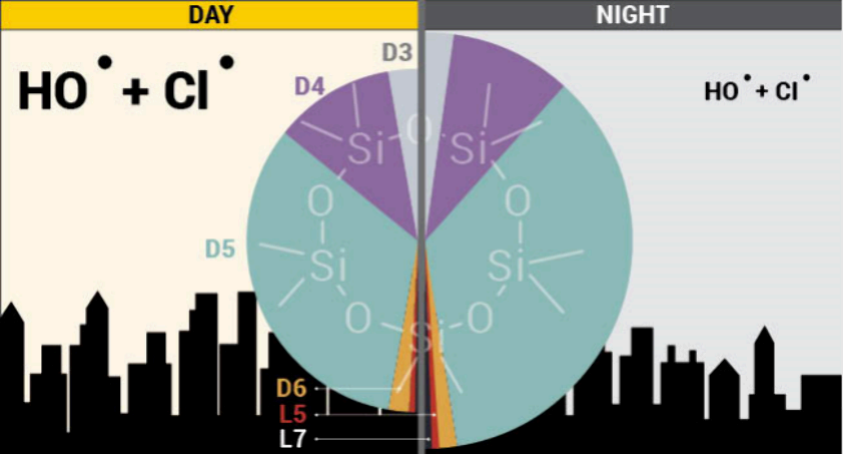

Volatile methyl siloxanes (VMS) are a group of organosilicon compounds of interest because of their potential health effects, their ability to form secondary organic aerosols, and their use as tracer compounds. VMS are emitted in the gas-phase from using consumer and personal care products, including deodorants, lotions, and hair conditioners. Because of this emission route, airborne concentrations are expected to increase with population density, although there are few studies in large urban centers. Here, we report summertime concentrations and daily variations of VMS congeners measured in New York City. Median concentrations of the 6 studied congeners, D3 (20 ng m^–3^), D4 (57 ng m^–3^), D5 (230 ng m^–3^), D6 (11 ng m^–3^), L5 (2.5 ng m^–3^), and L7 (1.3 ng m^–3^) are among the highest reported outdoor concentrations in the literature to date. Average congener ratios of D5:D4 and D5:D6 were consistent with previously reported emissions ratios, suggesting that concentrations were dominated by local emissions. Measured concentrations agree with previously published results from a Community Multiscale Air Quality model and support commonly accepted emissions rates for D4, D5, and D6 of 32.8, 135, and 6.1 mg per capita per day. Concentrations of D4, D5, D6, L5, and L7 and total VMS were significantly lower during the day than during the night, consistent with daytime oxidation reactivity. Concentrations of D3 did not show the same diurnal trend but exhibited a strong directional dependence, suggesting that it may be emitted by industrial point sources in the area rather than personal care product use. Concentrations of all congeners had large temporal variations but showed relatively weak relationships with wind speed, temperature, and mixing height.

## Abstract 2

Atmospheric concentrations of VMS in the atmosphere NYC were among the highest reported to date and exhibited significant variability primarily driven by diurnal changes in oxidative reactivity.

## Introduction

Volatile methyl siloxanes (VMS) are a group of synthetic chemical compounds defined by an organosilicon backbone with methyl functional groups attached at each silicon atom. Both cyclic (e.g., decamethylcyclopentasiloxane, [D5]) and linear (e.g., dodecamethylpentasiloxane [L5]) VMS congeners are valued in industrial and consumer applications because of their low reactivity, high stability, and low viscosity compared to chemicals with similar molecular weights.^1−3^ As a result, VMS have been heavily employed in the manufacture of consumer products such as adhesives, antifoaming agents, lubricants, and cleaners and are used as intermediates for polymeric silicones.^4−7^ VMS are particularly prevalent in personal care products (PCP) such as deodorant, shampoo, and skin cream where they are used as solvents or emollients.^1,8−10^ Several product surveys have reported that at least one VMS congener was present in upward of 90% of the commercially available personal care products tested.^1,8,9^ Today the global production of siloxanes is projected at over 2 million tons per year.^6,14^ Because of their high volatility and their ubiquity in personal care products, VMS are emitted into indoor air in large quantities resulting in concentrations in the range of 10,000 to 100,000 ng m^–3^. These indoor air concentrations are eventually released to the outdoors resulting in high concentrations near major population centers.^11,12^ VMS that are not released into indoor air are either directly emitted into the atmosphere off of consumer’s skin or are washed down the drain and subsequently emitted from wastewater treatment plants.^13−15^ As a result it is estimated that approximately 95% of VMS are eventually released into the atmosphere.^15^ Once released into the atmosphere, the primary degradation pathway for VMS is oxidation via reaction with hydroxyl and chlorine radicals.^6,16,17^ The oxidation rates of VMS are relatively slow in comparison to other common volatile chemicals leading to atmospheric lifetimes on the order of several days.^6,16−19^

Because of the scale of their production and their persistence in the atmosphere, there has been an increased focus in the past two decades on the fate and impact of VMS. Concerns have arisen about potential health effects of VMS such as endocrine disruption, decreased fertility, and lung and liver damage.^20−24^ Starting in 2020, the European Union enacted regulations limiting the use of two congeners, D4 and D5 to 0.1% w/w in wash-off cosmetics.^25^ While the use of VMS outside of the EU remains unrestricted, representatives from the personal care product industry have stated in public interviews that they believe manufacturers in the United States and other parts of the world are phasing out VMS in anticipation of future bans or to streamline the manufacture of products sold in both EU and non-EU markets.^26,27^ However, the real impact of these regulations on VMS emissions is still largely unknown. In addition, there has recently been increased attention on the potential for oxidized VMS byproducts to form secondary organic aerosols (SOA), a phenomenon which has been observed repeatedly in chamber studies.^28−31^ This SOA formation makes VMS potentially hazardous to urban air quality and may help to explain current gaps in the global SOA budget.^31^ VMS have also been proposed as tracer compounds for emissions from consumer products.^32−34^ Because of the significant interest in these compounds, there is a pressing need to better understand the concentrations of VMS in the atmosphere and the factors that control them.

A number of previous studies employing field measurements and atmospheric models have provided estimates of atmospheric VMS concentrations in areas ranging from remote parts of the arctic to major cities, with the majority of work to date occurring in the United States, Western Europe, or Eastern Asia.^3,11,12,34−48^ One trend consistently observed in these studies is that concentrations of all VMS congeners tend to be higher in densely populated urban areas.^3,11,35,36,47^ This is unsurprising given that the primary emissions source of VMS is personal care products and other volatile chemical products. Even though the emissions and concentrations of VMS tend to be very high in these areas, measurements of VMS concentrations in large cities remain scarce. To date in the literature, we found 59 unique nonindustrial study sites where VMS concentrations in outdoor air had been reported. Of these, only 7 had an average population density greater than 5000 capita per square kilometer within 20 km of the site. Furthermore, there has been relatively little comparison between VMS measurements and atmospheric models in these areas, making it difficult to assess the validity of these models near major emission sources. It is also worth noting that in many studies, measurements have been limited to 2 or 3 of the most abundant VMS congeners such as D4 and D5, leaving an incomplete picture of the total VMS concentration and less abundant congeners.^11,34,42,45^

While population density has been suggested as a strong predictor of average VMS concentrations, these concentrations typically exhibit large temporal variations on time scales as small as several hours. This is particularly true in urban settings where concentrations can change by a factor of 2 within a 12 h period.^11,44^ Factors such as temperature, wind speed, emissions timing, mixing height and hydroxyl radical concentrations have been shown to control VMS concentrations to varying degrees.^11,35,42,45^ However, the relative importance of these factors appears to differ between studies, and our ability to accurately predict the temporal variation of VMS, particularly in areas with high concentrations, is still limited.

To address these gaps, we conducted an approximately one-month long field sampling campaign in New York City, the most populated metro area in the United States both in terms of total population and population density. This field campaign measured 6 VMS congeners in air samples collected 3–5× per day during the months of July and August, which were subsequently analyzed via gas chromatography with triple quadrupole tandem mass spectrometry (GC-MS/MS) to determine their concentrations. We hypothesized that the concentrations of all VMS species in New York City would be among the highest measured to date and that these concentrations would be well predicted by the site’s population density and established per capita emissions rates for the United States. Furthermore, we hypothesized that temporal changes in the concentrations would be a function of an interplay of factors including meteorological conditions and the timing of PCP use.

## Methods

### Materials

Chemicals used in sample preparation and analysis can be found in Table S12. Details of standard and surrogate preparation can be found in the Supporting Information (p.27). Solid-phase extraction cartridges (EVOLUTE EXPRESS ABN, 600–0001-AXG, 10 mg sorbent, 1 mL volume) were purchased from Biotage Charlotte, NC. ABN cartridges were selected as previous work has demonstrated improved stability of VMS compounds on ABN media compared to ENV+ cartridges.^49^ Prior to field deployment, cartridges were soaked in hexane for at least 24 h. Cartridges were then removed from the hexane and allowed to fully dry. Cartridges were washed through 3 times with methylene chloride and then washed through 3 times with hexane. After allowing the cartridges to dry, they were individually wrapped in aluminum foil until field deployment.

### Field Site and Sampling Protocol

**Figure 1:**
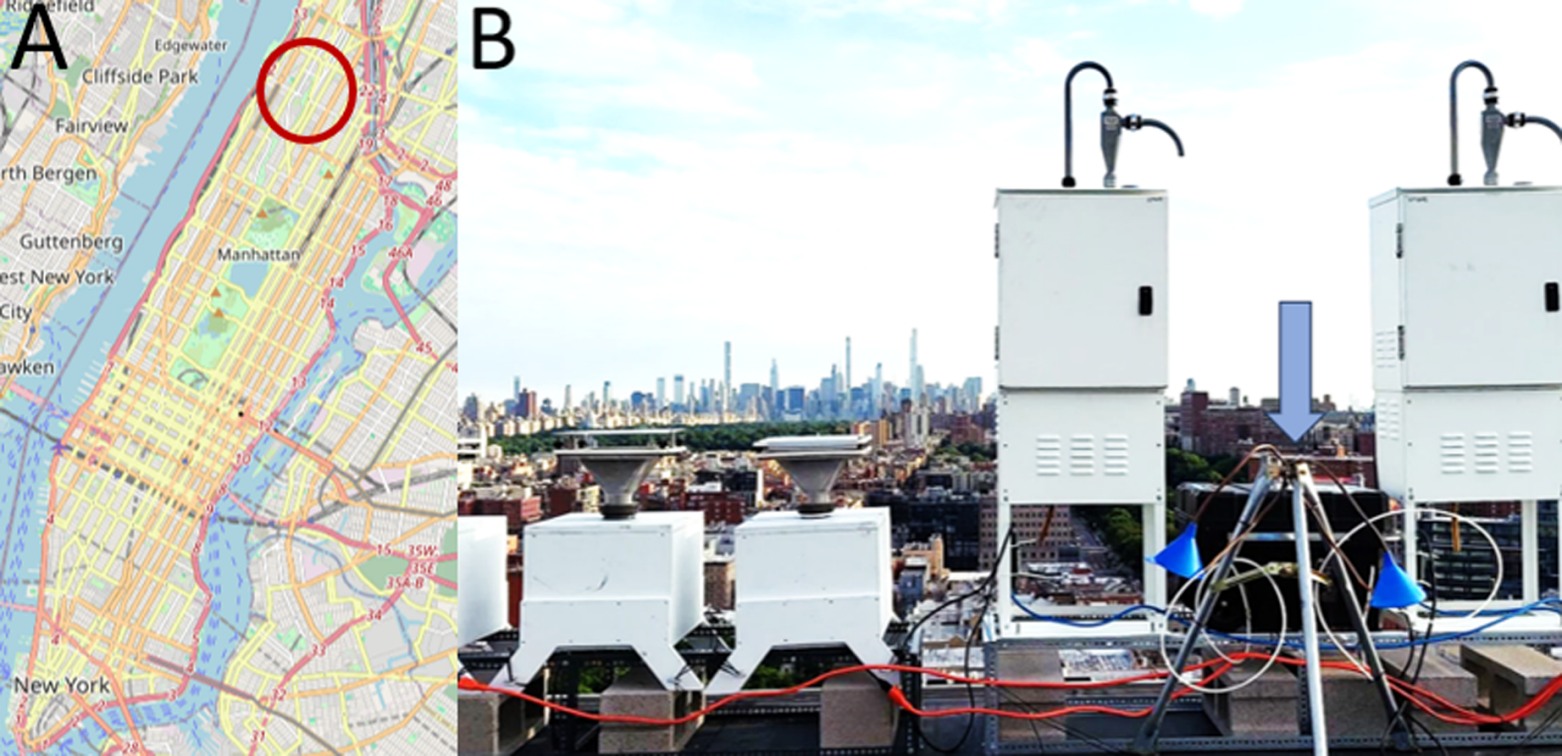
(A) Approximate location of sampling site in Manhattan, NYC. (B) Sampling equipment deployed on the roof of the ASRC. The samples described in this study were collected by low-volume samplers with the blue conical inlets in the right of the frame indicated by the blue arrow.

Samples were collected at the City University of New York’s Advanced Science Research Center (40.8153, −73.9504) hereafter referred to as “ASRC.” The ASRC is located in Harlem, a neighborhood of Manhattan, which is a borough of New York City, NY (Figure 1A). The primary land use in Harlem is residential consisting of multistory apartment buildings or mixed-use residential and commercial properties. Most of the land not occupied by residential or mixed properties consists of either purely commercial properties or public recreation areas such as parks or athletic facilities.^50^ A low-volume air sampler was constructed and deployed on the southwestern corner of the building’s roof approximately 40 m above the ground (Figure 1B). The sampler consisted of a GAST MAA-V109-HD oilless vacuum pump connected to the ABN cartridges with 1/4” PTFE tubing, which pulled air through the cartridges at a flow rate of 0.3 slpm. All junctions in the PTFE tubing and connections to the pump and cartridges were fitted with brass compression fittings. Cartridges were installed on the sampling system by fitting them into the compression fittings, sliding a section of 1/4” PTFE tubing over the connection and securing it with a hose clamp. Cartridges were installed facing downward and were shielded with plastic cones to protect from precipitation.

The sampler was separated from the building’s exhaust fans by a minimum of 25 m and from any doors and windows by a minimum of 10 m. Exhaust ports and lines from other sampling equipment were directed off the edge of the building and away from the sampler. Access to the roof site where samplers were deployed was strictly limited to personnel involved with the study. During the duration of the study, personnel who directly collected and handled samples refrained from all personal care product use. All other personnel on location were directed to avoid using personal care products prior to accessing the study site. Local wind direction, wind speed, and temperature data were obtained at 1 min resolution with a RM Young 81000 Sonic Anemometer deployed on the southeastern edge of the building within 10 m of the sampler. Temperature, wind speed, wind direction, and planetary boundary layer height (hereafter referred to as mixing height) data were obtained for the study site from the NOAA High-Resolution Rapid Refresh (HRRR) model archives (3 km, 1 h).^51^

During sample collection, samplers were run continuously except for when they were temporarily shut off for cartridge exchanges (∼5 min). As a result, collected samples represent a composite of the sampling duration during which they were deployed. Exchanges were performed directly on the roof and both the cartridges being collected and deployed were handled using clean nitrile gloves. Between 7/12/2022 and 7/26/2022 sample exchanges were performed 3 times per day to capture a “morning” sample (8:30–14:00), an “afternoon” sample (14:00–20:30), and an “overnight” sample (20:30–8:30). For the end of the study period between 7/27/2022 and 8/3/2022, sampling frequency was increased to 4 or 5 times per day. Times referenced above and in the rest of this work are in UTC-4 (EDT). Field blanks were collected approximately every 5 days by unwrapping two cleaned cartridges and installing them on samplers for the approximately 5 min period when no air was flowing between sample exchanges. Field blanks were then stored and transported in the same manner as the samples. Upon collection, ABN cartridges were wrapped in foil and transferred to a freezer. Samples were kept in freezer storage (−18 °C) for the duration of the field campaign until transported in a chilled sealed cooler to the University of Iowa. Samples were stored between 2 and 3 months before analysis.

### Sample Analysis and Quality Control

A multiple reaction monitoring (MRM) method for the detection of D3 (hexamethylcyclotrisiloxane), D4 (octamethylcyclotetrasiloxane), D5 (decamethylcyclopentasiloxane), D6 (dodecamethylcyclohexasiloxane), L5 (dodecamethylpentasiloxane), L7 (hexadecamethylheptasiloxane), surrogate standards (13C–D3, 13C–D4, 13C–D5, 13C–D6), and the internal standard (PCB-30) was developed for the Agilent 7000D GC-MS/MS Triple Quad (SI p.25–27, Table S13). ABN cartridges were removed from the freezer and allowed to come to room temperature. Prior to extraction, cartridges were spiked with surrogate standards (11.3 ng of 13C–D3, 12.3 ng of 13C–D4, 12.5 ng of 13C–D5, and 12.7 ng of 13C–D6). Cartridges were extracted with 1.5 mL of hexane directly into a 2 mL GC vial and then spiked with the internal standard (50 ng of PCB-30). Samples were sealed with PTFE crimp caps (Agilent, 5182–0871) and transferred to the instrument for analysis. A procedural blank sample was prepared with each run by spiking 1.5 mL of hexane with the same quantities of the surrogate standards and internal standard.

Quality control measures included rigorous procedures to prevent sampling and laboratory artifacts and the development of multiple methods for quantitative evaluation of quality assurance. To minimize contamination, the GC/MS Triple Quad was equipped with a Merlin Microseal and a low bleed Agilent DB-5ms column. Personal care products were not worn by lab personnel during extraction and analysis. All extracted samples were analyzed in duplicate to account for analytical variability. Breakthrough tests were conducted by sampling with two cartridges in series and both field and laboratory blanks were analyzed to determine background contamination (Figure S4). Average breakthrough to the secondary cartridge was less than 10% for all target compounds (Table S11). Analytical duplicates and field duplicates had an average relative percent difference (RPD) of 18% and 31%, respectively, across all samples and analytes (Table S8). L5 had higher variability than other compounds with an analytical and field RPD of 34% and 46%, respectively. Surrogate standard masses were quantified in all samples and procedural blank samples using the internal standard method. Surrogate recoveries were calculated as the surrogate mass in the sample divided by the surrogate mass in the procedural blank for the same analytical run. Surrogate recoveries were on average 88%, 88%, 94%, and 79% for 13C–D3, 13C–D4, 13C–D5, and 13C–D6, respectively, although select samples did exhibit recoveries as low as 50% or as high as 150% (Table S9). Field blanks showed low levels of all 6 congeners, indicating that on-site policies about PCP use were sufficient to avoid contamination (Table S10). Limits of quantification (the upper limit of the 99th confidence interval of the log-10 blank masses) were calculated as 2.325× the standard deviation of the log-transformed field blank masses plus their average and were found to be 2.1 ng (D3), 4.5 ng (D4), 12 ng (D5), 0.91 ng (D6), 0.34 ng (L5), and 0.05 ng (L7). The full data set of VMS concentration measurements has been released to an open-access data repository.^52^

### Data and Statistical Analysis

Population density data were obtained from worldpop.org from the “population density/unconstrained individual countries 2000–2020 UN adjusted (1km resolution)” data set.^53^ When determining local site population density, population density files from the year the samples were collected were used. If data were collected over multiple years, the latest year of the data set was used. For data collected after 2020, the 2020 data set was used. Population density for each location was calculated as follows. First, the straight-line distances between the measurement location and the centroid of all 1 km pixels within the relevant worldpop.org data set were calculated. Next, the population density was averaged for all pixels that fell within a given distance (e.g., 5 km). This was repeated for each study site at various distances as discussed later so that an average 1, 5, 10, 15, and 20 km population density was determined (Table S5). Meteorological data was paired to field measurements by averaging the values of all meteorological measurements which occurred during each composite sampling period (Table S2). The vector mean wind direction and standard deviation of the wind direction for each sampling period were calculated according to previously described methods using MatLab (R2022a).^54^ The mixing height for each sampling period was calculated as described previously by linearly interpolating the 1 h HRRR data to a 15 min time scale and calculating the inverse weighted mean of the data which occurred during each sampling.^11^ 95% confidence intervals for concentrations were calculated using a Monte Carlo analysis. For each iteration, a replicate mass was selected at random, and an air volume was obtained by sampling randomly from a probability distribution of the flow rate (0.3 ± 0.05 slpm) and multiplying it by the duration of the sampling period. A concentration value was then obtained for that iteration by dividing the replicate mass by the air volume. After 10,000 iterations, the 95% confidence interval was obtained by calculating the 2.5 and 97.5 percentiles of the distribution of concentrations obtained. This process was repeated individually for each VMS compound in each sampling period. Diurnal concentration patterns were determined by assigning the concentration measured in each sampling to 1 h periods of the day that fell within its sampling period (e.g., a sample collected between 8:00 and 12:00 was assigned to the hours 8, 9, 10, and 11). The median and 95% confidence intervals for each period were then calculated from the concentration measurements assigned to that hour. The statistical significance of linear regressions was evaluated with an F-test with *p* < 0.05 set as the cutoff for significance. In addition, the correlation and the significance of that correlation between variables was evaluated with a Pearson’s product moment correlation test with *p* < 0.05 set as the cutoff for significance. The normality of the log-transformed concentrations was assessed using the Shapiro–Wilk test and all congeners were normally distributed (*p* < 0.05). Statistical analysis was performed in RStudio (2023.06.2, Posit Software PBC). Data visualization was performed using R packages ggplot2 and Openair.^55,56^

## Results and Discussion

### VMS Concentrations, Ratios, and Correlations

**Figure 2:**
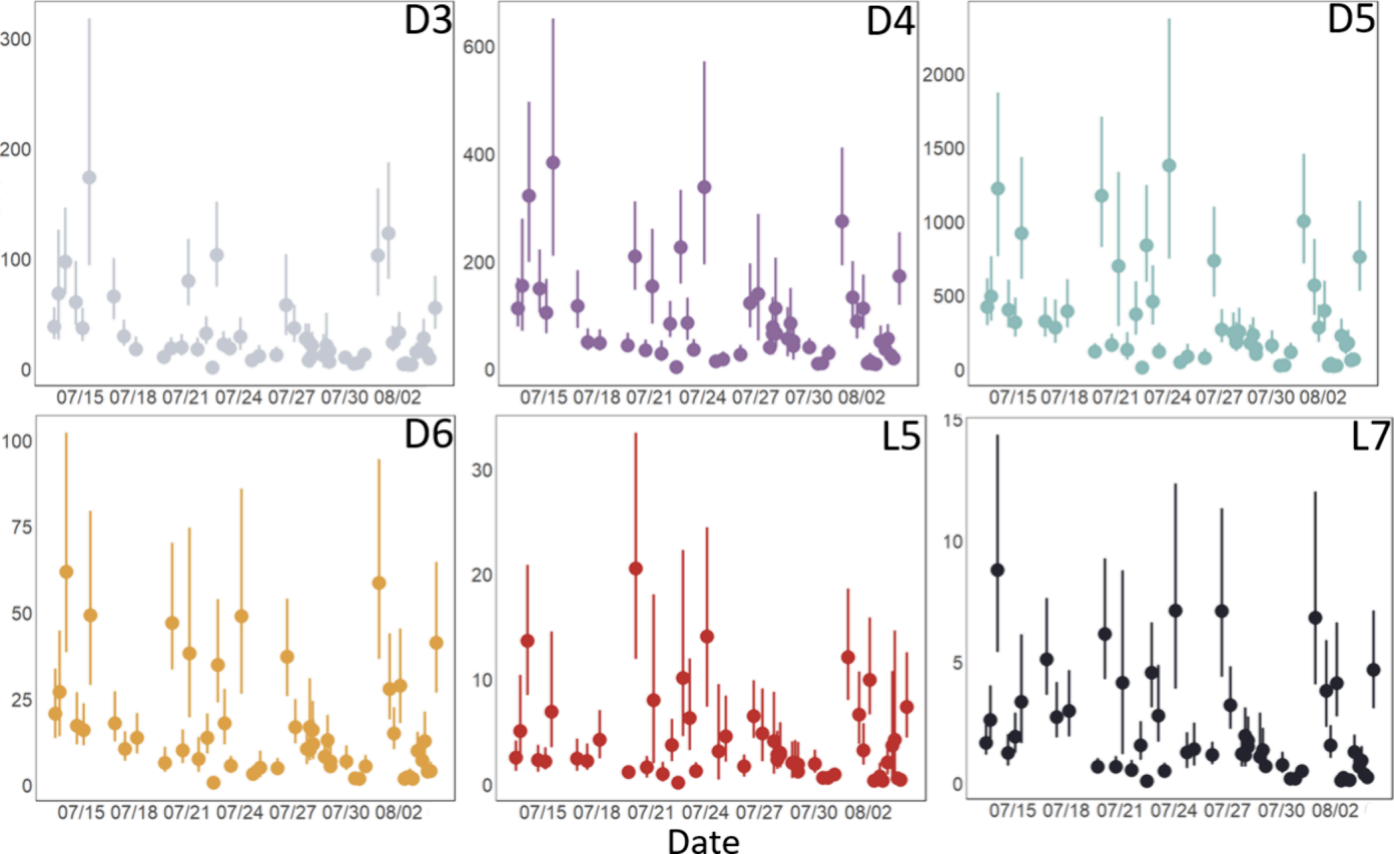
Concentration (ng m^–3^) time series of D3, D4, D5, D6, L5, and L7 between 7/13/2022 and 8/4/2022. Center dots represent the concentration (average of the two field duplicates) at each time point and bars represent the 95% confidence interval based on a Monte Carlo analysis (see methods). Note variation in *y*-axes. Data points are displayed on the *x*-axis at the midpoint of the composite sampling period.

The median of the total VMS concentration (sum of the 6 measured congeners) for the study period was 290 ng m^–3^ and ranged between 19 and 1800 ng m^–3^. Concentrations of all congeners (D3, D4, D5, D6, L5, and L7) between the period 7/13/2022 and 8/4/2022 were among the highest outdoor concentrations reported in the literature to date (Figure 2). Full summary statistics for individual congeners are in Table 1. Our study does not include all possible VMS congeners, but we consider these numbers to be representative of the “total VMS” concentration given the low abundance of other congeners observed in previous studies. For example, in measurements from four sites in urban Japan, which included D3–D9 and L3–L15, the 14 congeners not included in our measurements constituted only 4% of the total VMS mass.^35^

**Table 1:** Summary Statistics of Individual VMS Concentrations

	D3 (ng m^–3^)	D4 (ng m^–3^)	D5 (ng m^–3^)	D6 (ng m^–3^)	L5 (ng m^–3^)	L7 (ng m^–3^)
median	20	57	230	11	2.5	1.3
min	1.5	3.6	13	0.70	0.20	0.10
maximum	170	380	1400	62	21	8.8
IQR	11–37	29–120	110–420	5.5–20	1.2–5.1	0.70–3.2

Concentrations of D5 were higher than any other congener for the entire study period. Of the measured congeners, D5 constituted 70% of the total airborne VMS concentration. Following D5, D4 was measured at the next highest concentration followed by D3, D6, L5, and then L7. This concentration pattern is consistent with previous indoor and outdoor air measurements where D5 has been universally observed as the highest concentration congener.^3,11,35,36^ Concentrations of both L5 and L7 were an order of magnitude below the concentrations of the cyclic species consistent with a number of previous studies which have shown that linear VMS as a whole typically represent less than 5% of the total airborne VMS mass.^36,41,44,47^ Average congener concentrations ratios (Table 2) were comparable to those reported in previous measurements. In particular, the average ratios of D5:D4 (3.6) and D5:D6 (19) were not significantly different (unpaired *t* test, *p* < 0.05) from those reported in measurements from Chicago (4.5 and 20 respectively), which were later determined to be within 1% of the ratios present in fresh emissions based on photochemical age.^11,12^ The D5:D4 ratio from our study is also consistent with that predicted by the United Kingdom’s Environment Agency for emissions from “use by the general public” of 3.0.^57,58^ The similarity between these ratios suggests that VMS concentrations in New York City are dominated by local emissions and are minimally influenced by long-range transport from other cities or industrial sites.

**Table 2:** (Top Right (**Bolded**)) Median Ratios (Column Heading/Row Heading) of Average Congener Concentrations in New York City Air Samples and (Bottom Left (*Italicized*)) Correlations between the Log-Transformed Concentrations of VMS Congeners^a^

	D3	D4	D5	D6	L5	L7
D3	1.0	**2.6**	**8.6**	**0.45**	**0.12**	**0.06**
D4	*0.88*	1.0	**3.4**	**0.05**	**0.04**	**0.02**
D5	*0.88*	*0.96*	1.0	**0.05**	**0.01**	**0.01**
D6	*0.89*	*0.97*	*0.98*	1.0	**0.22**	**0.11**
L5	*0.77*	*0.86*	*0.90*	*0.91*	1.0	**0.52**
L7	*0.84*	*0.90*	*0.93*	*0.93*	*0.93*	1.0

a All correlations were statistically significant (*p* < 0.05, Pearson’s product moment correlation test).

The log-transformed concentrations of all congeners were significantly correlated with each other (Table 2). D3 was the least correlated with other congeners with an average correlation coefficient of 0.85. The lowest correlation coefficient between any two congeners was between D3 and L5 (ρ = 0.77). D5 and D6 were the most strongly correlated with other congeners (average ρ= 0.93) and were the two most strongly correlated individual congeners (ρ = 0.98). The strong correlation between D5 and D6 is likely a result of their common emissions sources as similar strong correlations have been observed in direct measurements of U.S. personal care products.^9^ However, it is worth noting that this same relationship between D5 and D6 has not been observed in product measurements from other countries and, in general, the makeup of VMS in consumer products has both changed over time and varies greatly between countries.^1,5,8,9^ As a result, these correlations can only be considered reflective of the mix of VMS sources present in the United States at this point in time.

### Comparison to Previous Studies and Population Density

**Figure 3:**
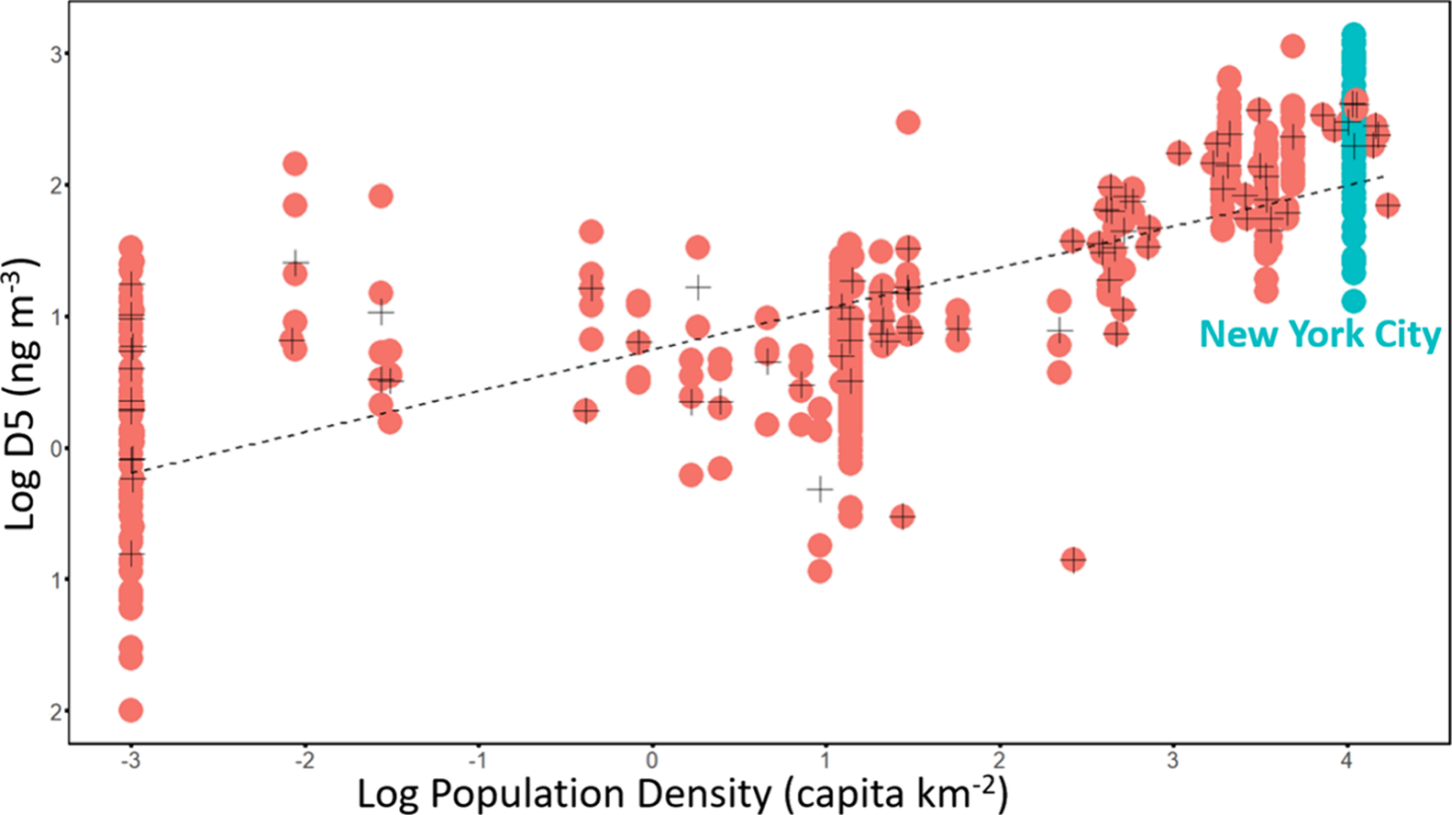
Log D5 concentrations as a function of the log-transformed population density within 10 km of the study site from our study and as reported in previous literature between 2000 and present, in areas with no local manufacturing. Individual measurements are shown as solid dots while the central tendencies of the sites are shown as crosses. If multiple data points were available for a site, the mean of the log-transformed values was used as the central tendency. If only a single data point or an average was reported from a previous study this was log-transformed and used as the closest approximation of the central tendency. Previous measurements are shown in red while data from our study is shown in teal. A dashed line denotes the line of best fit for the linear regression.

Our measurements provide further evidence that concentrations of D5 are well predicted by population density and that per capita emissions of D5 in Western countries remain at levels consistent with data sampled during the period (2000-present). Data from our study site when combined with previous data in the literature show that there is a strong and statistically significant relationship (*R*^2^ = 0.59, *p* < 0.001) between log D5 concentrations and the log of average population density within 10 km of the location in which the measurements were conducted (Figure 3, Figure S3).^3,11,34−39,42−46,48,49,59^ We selected 10 km because it was the strongest predictor out of the distances we tested between 1 and 20 km. Furthermore, the agreement between our data and the historical trend shows that the per capita emission rate of D5 in the United States has not significantly declined despite industry suggestions that EU restrictions may be causing a voluntary phase out of VMS in U.S. manufacturing.^26,27^ However, comparisons between our data and previous VMS measurements also show that the relationship between concentration and population density is not as strong for all VMS congeners. The relationships between log population density and log D6 (*R*^2^ = 0.55), log D4 (*R*^2^ = 0.32), and log D3 (*R*^2^ = 0.28) were much weaker than for D5 (Table S7). These weaker relationships indicate that non-PCP volatile chemical products may significantly contribute to the emissions of D3, D4, and D6 and highlight the complexity of predicting total atmospheric VMS concentrations. L5 and L7 could not be evaluated because of the lack of available data, particularly in large cities. It must be noted that even for D5, population density can only be considered a rough proxy, and other factors such as per capita PCP usage, local geography, and point source emissions (i.e., silicone manufacturing, PCP formulation, wastewater treatment, and landfills) must be considered when predicting VMS concentrations.

**Figure 4:**
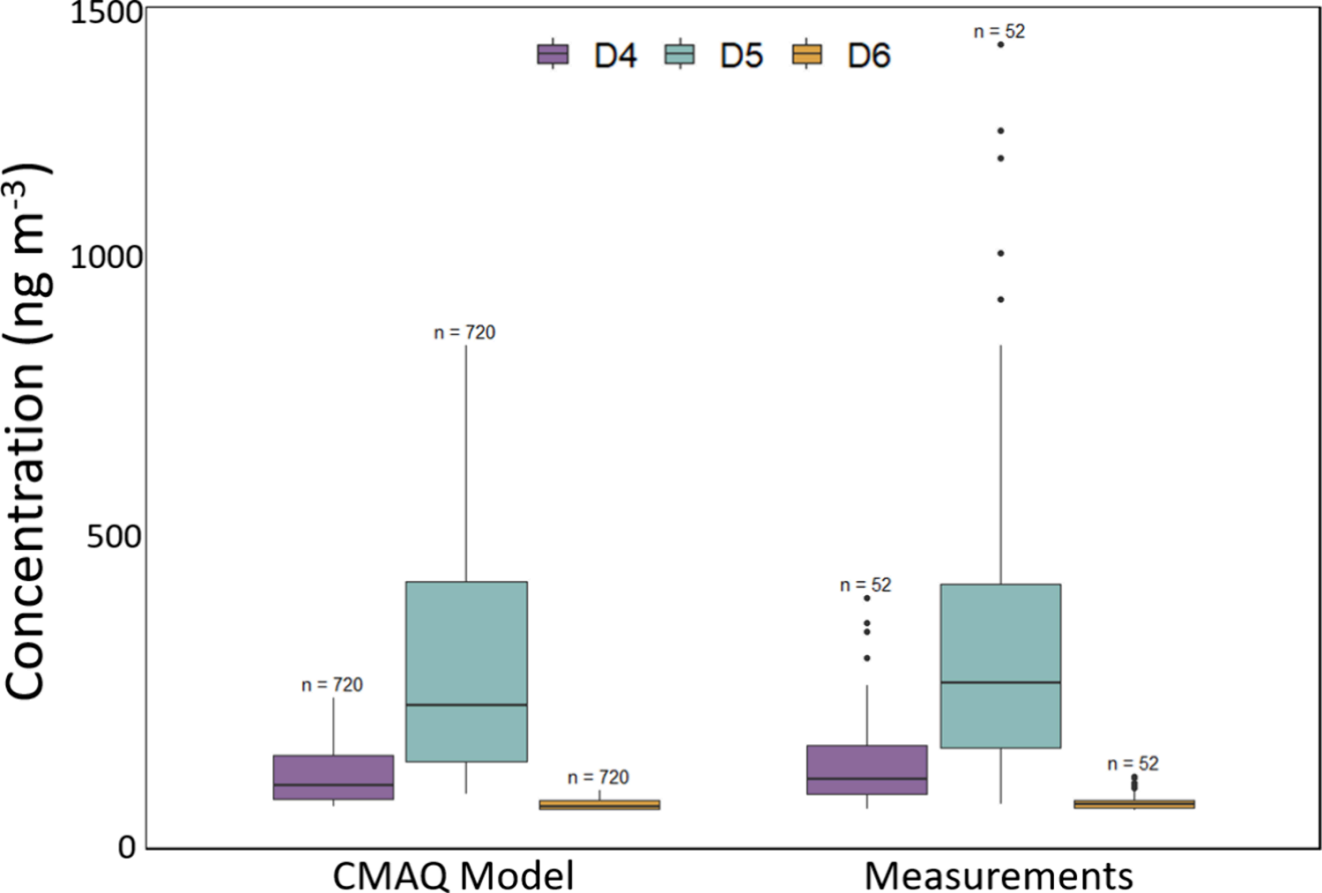
Atmospheric concentrations of the VMS congeners D4, D5, and D6 predicted by the Janechek et al. 2017 CMAQ model for the month of July compared to measurements of the same congeners from our study period (July, August). The center line of each box represents the median value while the box contains the 25th and 75th percentile and whiskers denote inside middle 95% of the data.

We compared our measurements to previously published results from a Community Multiscale Air Quality model (CMAQ) of D4, D5, and D6.^12^ Our comparison is the first validation to date of a VMS chemical-transport model in an area with the extreme population density (and VMS emissions) of NYC. For the purposes of this comparison, we compiled D4, D5, and D6 concentrations from the model’s previously published data set.^60^ This CMAQ model utilized a 36 km horizontal resolution with one grid cell that captured the whole of the New York City metro area. From this grid cell, we compared data from 720 h of the “July” model run to our measured concentrations (which occurred between July and early August). Although the published model results represent a prior year (and consequently it is impossible to compare the concentration time series to our results), it is still possible to compare the average model concentrations (and by extension the per capita emissions estimates) to our measurements since the population of New York City has only increased 4% between 2011 (the simulated emissions year) and the time we conducted our study (2022).^61^ The concentrations of all three VMS species in our measurements appeared to be well predicted by the CMAQ model with a small degree of underestimation (Figure 4). Median concentrations from the model output were 20% (D4), 19% (D5), and 27% (D6) lower than our measured concentrations and the log-transformed means were not statistically different (unpaired *t* test, *p* < 0.05). In fact, the agreement may even be stronger than these results indicate since the model output represents an average of the whole NYC metro area while our study site is located in the most densely populated area of the city and thus the measurement site may not be representative of the model grid average (with high bias). This comparison supports the idea that the daily per capita emission rates of D4 (32.8 mg), D5 (135 mg), and D6 (6.1 mg) used in this model would also be suitable for modeling 2022 emissions in the United States. However, future comparisons across a variety of geographies combined with measurements of other known markers of PCP and other emissions sources is needed to provide full validation of these emissions rates. In addition, this comparison provides further confirmation that urban VMS emissions in the United States have not noticeably declined as of 2022 since atmospheric concentrations are still well predicted by estimates made using 2011 emissions.

### Relationship with Meteorological Variables

Concentrations of all 6 congeners exhibited large temporal variations throughout the sampling campaign with relative standard deviations between 94% (D6) and 107% (D3). Total VMS concentrations changed by more than a factor of 5 within 12 h multiple times during the study period. However, this variation does not seem to be well predicted by meteorological variables relative to reports from previous studies. Regressions between the natural log of VMS concentrations and wind speed follow the expected direction (fast wind speeds driving lower concentrations); however, these relationships were either relatively weak [D4 (*R*^2^ = 0.10), D5 (*R*^2^ = 0.09), D6 (*R*^2^ = 0.08)] or were not significant at all [D3, L5, L7] (Table S3, Figure S1).^44^ Mixing height regressed against natural log of VMS concentrations also followed the anticipated direction (lower concentrations at higher mixing heights). While these relationships were significant for all VMS species except for D3, they were also relatively weak with *R*^2^ values between 0.18 [D4] and 0.20 [D5] (Table S3, Figure S1). One possible explanation for the lack of strong relationships is that VMS emissions in this area are too large to become well mixed within the mixed layer. As noted previously, our site in Harlem is one of the most densely populated areas studied to date. While the average population density (capita per square km) within the broad NYC area is already high at 7000, within 1 km of the study site this density approaches 30,000. The high rate of PCP usage in the United States coupled with this extreme population density may create emissions large enough to produce vertical concentration gradients during periods when other species are well mixed. This vertical concentration gradient in turn may mitigate the effects of wind and mixing height on VMS. This phenomenon has been previously observed in measurements of CO and NO_*x*_ from traffic emissions where strong vertical profiles developed even during unstable meteorological conditions.^62−64^ Windspeeds may also have had less of an effect on VMS concentrations than observed in previous studies due to the fact that population density does not decline substantially within several kilometers of our study site. In cities such as Toronto where a strong windspeed effect has been previously observed, there is a small several kilometer area with high population density (and thus large VMS concentrations) which are then diluted by lower concentration air parcels during periods with high windspeeds. However, at our study site, it is likely that high concentration air mostly mixes with other high concentration air since population density remains well above 30,000 capita per km^–2^ within several kilometers. Clausius–Clapeyron plots also show weak relationships between concentrations and temperature. In fact, for all analytes, these relationships show the opposite of volatilization effect with higher concentrations occurring at low temperatures (Table S3, Figure S1). This reverse trend may be related to the relationship between temperature and the concentrations of oxidizing species as discussed below.

### Diurnal Trend and Directional Dependence

**Figure 5:**
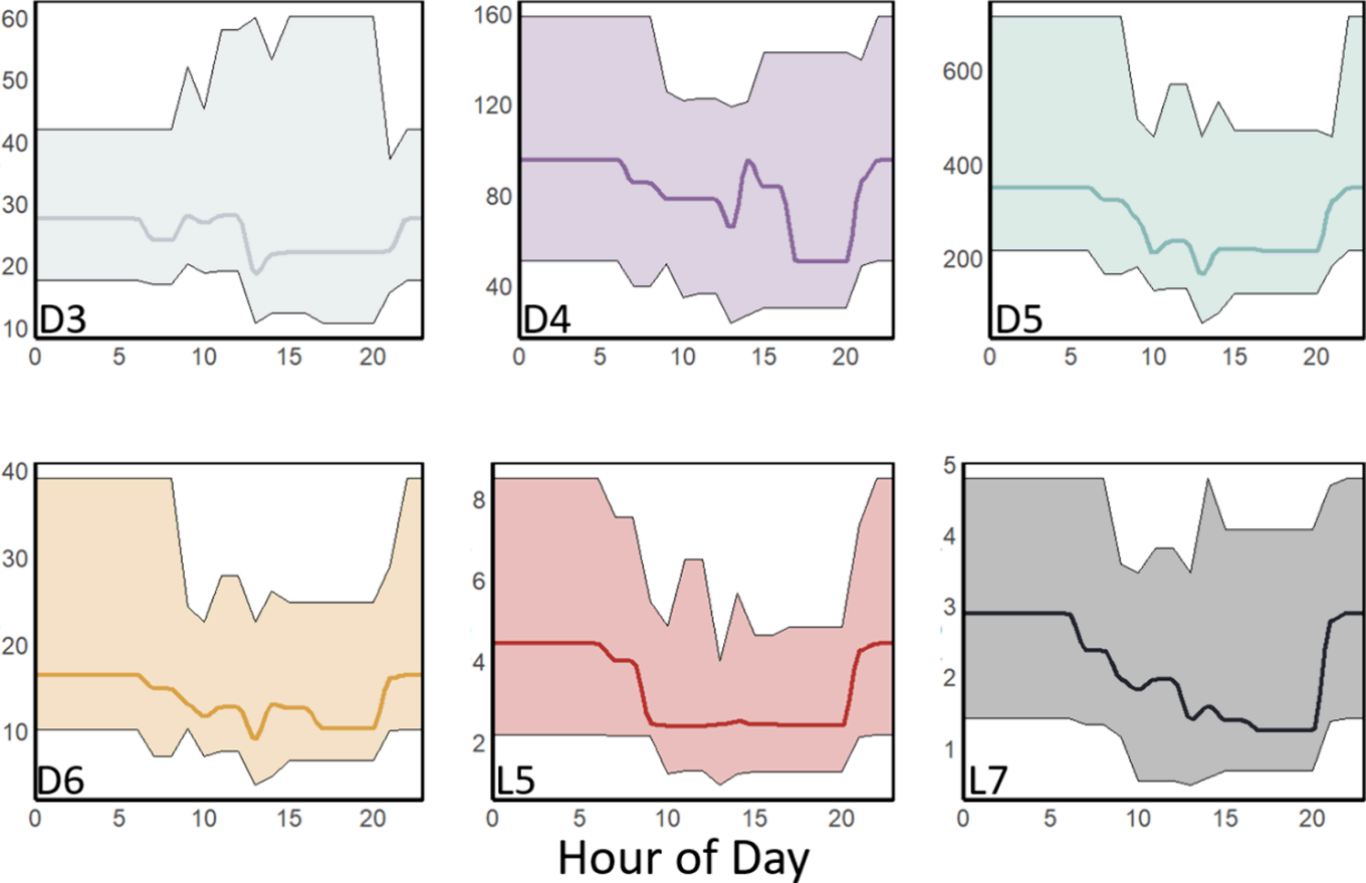
Diurnal concentrations (ng m^–3^) of D3, D4, D5, D6, L5, and L7 in New York City air samples for the duration of the study period. The solid line indicates the median concentration for each hour of the day while the shaded region denotes the interquartile range. Note that these numbers are based on averaging times of 6–8 h and do not necessarily reflect the precise ranges for 1 h period.

Concentrations of almost all VMS congeners exhibited a strong diurnal trend. Mean log-transformed concentrations of D4, D5, D6, L5, and L7 and total VMS were significantly higher (unpaired *t* test, *p* < 0.05) during the “nighttime” [21:00–9:00] than during the “daytime” [9:00–21:00] (Figure 5, Table S4). These cutoffs for “day” and “night” were selected as the first and last cartridge exchanges of the day and typically occurred around 8:45 and 20:45. It is possible that some of this diurnal trend may be driven by changes in the mixing height as observed in previous urban settings.^11^ However, as we have already shown, the relationships between VMS concentrations and mixing height are relatively weak and do not fully account for the 37% difference between the median “day” and “night” VMS concentrations. We speculate that daytime oxidation also plays an important role in the diurnal trend. Concentrations of hydroxyl radicals (the primary loss pathway for VMS) are elevated during the daytime, particularly in urban areas, and typically peak around noon.^18,65,66^ This diurnal oxidative effect has been previously simulated in compartmental models of urban VMS concentrations with sensitivities large enough to explain the observed decline in our measurements given New York City’s typical hydroxyl radical concentrations.^18^ Furthermore, this oxidative effect may be enhanced in New York City by chlorine oxidation. Although chlorine is estimated to contribute to a relatively small (5%) of global VMS loss, it may be far more important in urban settings with elevated chlorine radical concentrations.^6^ In contrast to previously studied inland cities in North America (Chicago, Boulder, Toronto), coastal New York City is predicted to have elevated concentrations of tropospheric chlorine, which may enhance this oxidative effect and explain why oxidation appears to play a more important role in our measurements.^67^ We note that because of the temporal resolution of our measurements we were not able to observe the previously reported early morning peak in urban D5 concentrations due to PCP use.^45^

In contrast to the other VMS congeners, median concentrations of D3 did not exhibit significant diurnal differences (Figure 5, Table S4). This may be related to the slower oxidation rate of D3 compared to the other VMS congeners included in this study, which should reduce the influence of daytime increases in oxidizing species.^6^ This trend may also be related to non-PCP emissions sources that remain elevated throughout the day (as opposed to PCP emissions which typically peak in the early morning and evening).^4^ Direct measurements of D3 in consumer products are limited but typically result in either very low concentration measurements or nondetects.^68^ In one survey of Canadian cosmetics, D3 was detected in only 0.79% of the tested products.^5^ These low levels do not explain the observed atmospheric concentrations of D3, which are greater than D6, L5, and L7, suggesting an alternate source. D3 was also recently reported as the predominant VMS congener present in vehicular emissions (particularly those of heavy duty vehicles).^69^

**Figure 6:**
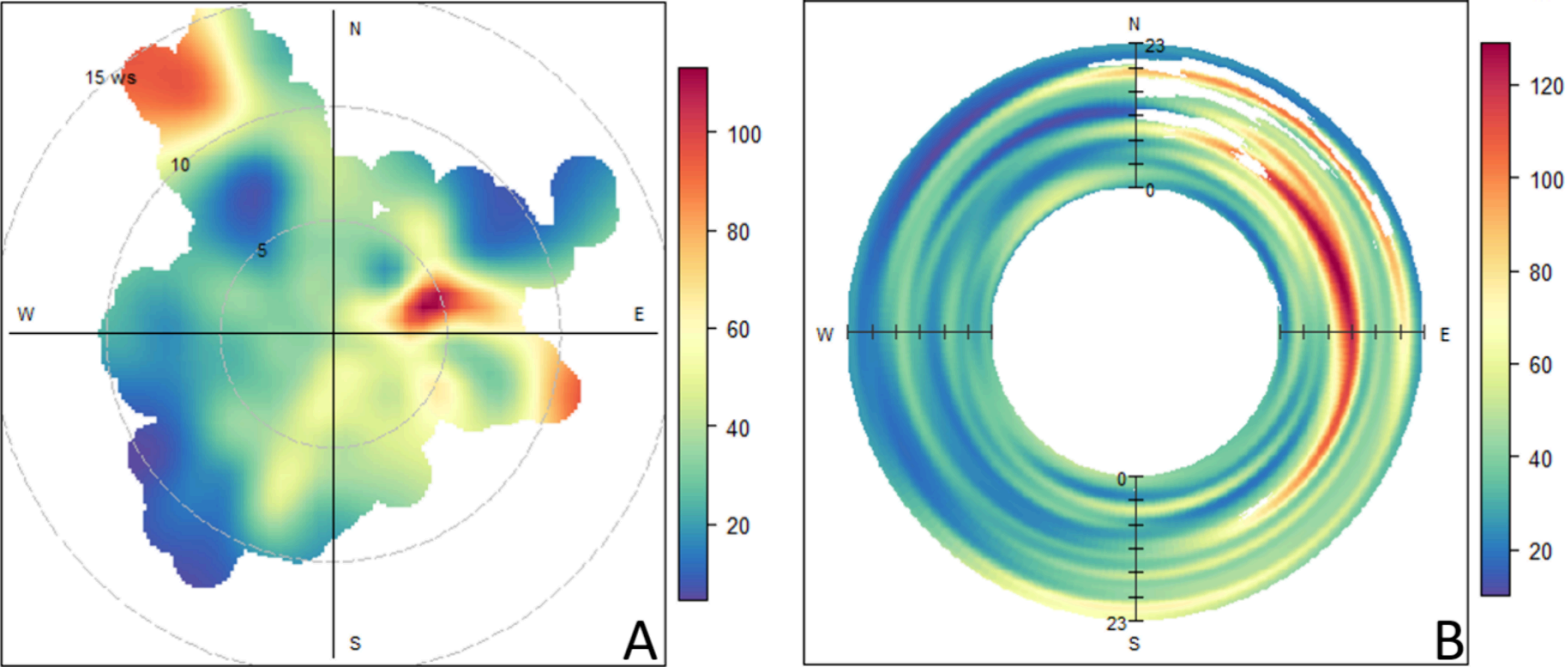
(A) Bivariate polar plot of D3 concentrations (ng m^–3^) as a function of windspeed (m s^–1^) and wind direction. (B) Bivariate polar plot of D3 concentrations (ng m^–3^) as a function of wind direction and time of day.

Furthermore, changes in the D3 concentration were significantly correlated (*p* < 0.05, Pearson correlation test) with the degree of change in wind direction, indicating that it may be emitted by local sources. Bivariate polar plots of D3 show that elevated concentrations occur during periods with slow easterly winds and that elevated daytime concentrations only occur with these easterly winds (Figure 6). Based on the location of our study site, we surmise that this may indicate air mass transport from southern Brooklyn, an area which is primarily zoned as an M3 district for pollutant generating industries such as solid waste transfer and recycling.^70^ Elevated concentrations also appear to occur when there are strong northwesterly winds, a phenomenon which cannot be as readily explained. Still, the dependence of D3 on wind direction in addition to its lack of a diurnal trend, and its absence in PCP measurements, lead us to believe that D3 may primarily be emitted in NYC by industrial and manufacturing sources. These alternate emissions sources would also explain why D3 concentrations are not as strongly correlated with other VMS congeners and why D3 concentrations are not predicted well by population density.

## Data Availability

The data underlying this study are openly available in Iowa Research Online at DOI: https://doi.org/10.25820/data.006807

## Supporting Information Available

Additional information including locations and population densities of previous field studies, additional population density regressions, additional meteorological data and regressions, detailed study site and sampling information, detailed QA/QC results, and additional GC/MS method details (PDF)
