# Supplementary material for: Concentrations of Volatile Methyl Siloxanes in New York City Reflect Emissions from Personal Care and Industrial Use
Source: Environ Sci Technol. 2024 May 9;58(20):8835–45. doi: 10.1021/acs.est.3c10752 (PMC11112754; doi:10.1021/acs.est.3c10752)
Supplement: Supplementary file 1 — es3c10752_si_001.pdf [file es3c10752_si_001.pdf]

Supporting Information

Concentrations of Volatile Methyl Siloxanes in New York City Reflect Emissions from Personal Care and Industrial Use.

Authors: Christopher E. Brunet[a], Rachel F. Marek[a], Charles O. Stanier[b] Keri C. Hornbuckle[a]*

[a]*Department of Civil and Environmental Engineering, IIHR-Hydroscience & Engineering University of Iowa, Iowa City, Iowa 52242, United States*

[b]*Department of Chemical and Biochemical Engineering, IIHR-Hydroscience and Engineering, The University of Iowa, Iowa City, Iowa 52242, United States*

*Email: keri-hornbuckle@uiowa.edu

Data generated in this research are available at: DOI: 10.25820/data.006807

Number of pages: 28

Number of tables: 13

Number of figures: 4

Contents

Study Site and Sampling Information........3

Table S1: Sampling periods, collected air volume, and measured concentrations of VMS congeners for collected samples........3

Meteorological Data Collection, Validation, and Analysis........6

Table S2: Meteorological data from on-site anemometer and NOAA High-Resolution Rapid Refresh model averaged for sampling periods........6

Table S3: R^2 and p-values (listed in parentheses) for linear regressions between VMS concentrations (and natural logs of VMS concentrations) and meteorological variables........8

Table S4: Median and Mean Log Transformed VMS concentrations for “Day” and “Night”........8

Figure S1: Linear regressions between the natural log of D3 (1), D4 (2), D5 (3), D6 (4), L5 (5), and L7 (6) concentrations and windspeed (A), mixing height (B), and inverse temperature (C)........9

Figure S2: Bivariate polar plots of D3 (A), D4 (B), D5 (C), D6 (D), L5 (E), and L7 (F) concentrations (ng m^{-3}) as a function of windspeed and direction........10

Previous Study Locations, Population Density, and Regressions........10

Table S5: Selected Sites, Sampling Method, and Population Densities........11

Table S6: Log D5 Concentration vs Log Population Density Regression Values........15

Table S7: Log VMS Concentration vs Log Population Density Regression Values........15

Figure S3: Regressions between Log D3 (A), D4 (B), D4 (C), and D6 (D) and Log Population Density........16

Quality Assurance and Control........16

Table S8: Average and standard deviation of relative percent differences (RPD) between analytical and field duplicates collected during the NYC sampling campaign........17

Table S9: Surrogate standard recoveries for all analyzed samples........17

Table S10: Field Blank Masses and Limits of Quantification........23

Figure S4: Setup of cartridges for breakthrough tests........24

Table S11: Calculated Breakthrough Percentages........24

Method Development, Instrument Parameters, and Standard Preperation........25

Table S12: Chemicals In Use........25

Table S13: Precursor Ion, Product Ions, Elution Times, and Collision Energies for Target Analytes........27

References........28

Study Site and Sampling Information

Table S1: Sampling periods, collected air volume, and measured concentrations of VMS congeners for collected samples.

Sample #	Sampling Start Time (EDT)	Sampling End Time (EDT)	Sample Volume (L)	D3 (ng m^{-3})	D4 (ng m^{-3})	D5 (ng m^{-3})	D6 (ng m^{-3})	L5 (ng m^{-3})	L7 (ng m^{-3})
1	7/13/2022 8:46	7/13/2022 14:10	97	38	110	420	21	2.6	1.7
2	7/13/2022 14:17	7/13/2022 20:20	110	69	160	490	27	5.1	2.6
3	7/13/2022 20:58	7/14/2022 8:39	210	97	320	1200	62	14	8.8
4	7/14/2022 14:05	7/14/2022 20:31	120	61	150	400	17	2.3	1.3
5	7/14/2022 20:36	7/15/2022 8:37	220	37	100	320	16	2.2	1.9
6	7/15/2022 8:40	7/15/2022 13:52	94	170	380	920	49	6.9	3.4
7	7/16/2022 8:45	7/17/2022 8:39	430	66	120	330	18	2.5	5.1
8	7/17/2022 8:44	7/17/2022 12:20	65	30	50	280	11	2.2	2.7
9	7/17/2022 20:46	7/18/2022 9:31	230	18	50	390	14	4.3	3.0
10	7/19/2022 14:15	7/19/2022 20:31	110	11	44	120	6.5	1.2	0.68
11	7/19/2022 20:39	7/20/2022 8:47	220	19	210	1200	47	21	6.2
12	7/20/2022 14:27	7/20/2022 20:15	110	20	35	160	10	1.6	0.68
13	7/20/2022 21:03	7/21/2022 8:24	210	80	150	700	38	8.0	4.1
14	7/21/2022 8:55	7/21/2022 20:30	210	18.0	28	130	7.6	1.0	0.53

15	7/21/2022 21:03	7/22/2022 8:38	210	32	84	370	14	3.7	1.6
16	7/22/2022 8:56	7/22/2022 13:48	88	1.5	3.6	13	0.74	0.16	0.10
17	7/22/2022 13:59	7/22/2022 20:30	120	103	230	840	35	10	4.6
18	7/22/2022 20:36	7/23/2022 8:24	210	22	86	460	18	6.3	2.8
19	7/23/2022 8:36	7/23/2022 13:29	90	19	37	120	5.6	1.3	0.51
20	7/23/2022 13:37	7/24/2022 12:44	420	30	340	1400	49	14	7.1
21	7/24/2022 12:49	7/24/2022 20:36	140	8.2	15	48	3.2	3.2	1.3
22	7/24/2022 20:40	7/25/2022 8:30	210	12	18	86	5.1	4.6	1.4
23	7/25/2022 20:35	7/26/2022 8:36	220	13	28	78	4.9	1.7	1.2
24	7/26/2022 10:44	7/26/2022 20:45	180	58	122	740	37	6.5	7.1
25	7/26/2022 20:49	7/27/2022 8:33	210	37	140	270	17	4.9	3.2
26	7/27/2022 16:19	7/27/2022 20:29	75	27	41	250	11	4.1	1.2
27	7/27/2022 8:36	7/28/2022 12:22	500	26	78	180	10	2.4	2.0
28	7/27/2022 8:52	7/28/2022 12:29	500	8.0	65	200	17	2.8	1.2
29	7/27/2022 12:34	7/28/2022 16:11	500	12	110	250	16	2.9	1.5
30	7/27/2022 20:33	7/28/2022 8:47	220	21	67	260	12	3.0	1.8
31	7/28/2022 16:16	7/28/2022 20:25	75	12	56	180	8.3	2.1	1.1
32	7/28/2022 8:28	7/29/2022 12:20	500	22	85	230	13	2.1	1.4

33	7/28/2022 12:25	7/29/2022 16:10	500	17	52	140	6.9	1.9	0.71
34	7/28/2022 20:28	7/29/2022 8:24	220	6.8	44	100	5.5	1.2	0.70
35	7/29/2022 16:14	7/30/2022 8:38	300	11	40	160	6.8	2.0	0.76
36	7/30/2022 8:43	7/30/2022 14:44	110	4.9	9.8	27	2.0	0.61	0.18
37	7/30/2022 14:49	7/30/2022 20:34	100	5.9	11	27	1.9	0.65	0.20
38	7/30/2022 20:38	7/31/2022 8:23	210	13	30	120	5.5	0.95	0.52
39	7/31/2022 8:30	8/1/2022 8:35	440	103	270	1000	59	12	6.8
40	8/1/2022 8:40	8/1/2022 14:13	100	120	130	570	28	6.7	3.8
41	8/1/2022 14:16	8/1/2022 20:24	110	24	89	280	15	3.3	1.6
42	8/1/2022 20:29	8/2/2022 6:26	180	33	110	400	29	10	4.1
43	8/2/2022 6:31	8/2/2022 8:27	35	4.3	11	25	1.9	0.36	0.12
44	8/2/2022 8:31	8/2/2022 12:34	73	5.3	15	40	2.4	0.45	0.22
45	8/2/2022 12:40	8/2/2022 16:37	71	3.9	9.9	21	2.4	0.75	0.16
46	8/2/2022 16:40	8/2/2022 20:38	72	4.2	9.5	25	1.9	0.36	0.14
47	8/2/2022 20:33	8/3/2022 6:15	180	16	51	230	10	2.1	1.3
48	8/3/2022 6:26	8/3/2022 8:28	37	19	37	160	7.2	3.7	0.68
49	8/3/2022 8:34	8/3/2022 12:43	75	28	57	180	13	4.3	0.95
50	8/3/2022 12:48	8/3/2022 16:50	73	15	26	63	4.0	0.59	0.36

51	8/3/2022 16:55	8/3/2022 20:24	63	9.8	20	69	4.1	0.46	0.25
52	8/3/2022 20:31	8/4/2022 8:20	210	56	170	760	41	7.4	4.7

Meteorological Data Collection, Validation, and Analysis.

On-site temperature, windspeed, and wind direction data were obtained at a 1-minute resolution with a RM Young 81000 Sonic Anemometer. Additionally, temperature, wind speed, wind direction, and planetary boundary layer height (hereafter referred to as mixing height) data were obtained for the study site from the NOAA High-Resolution Rapid Refresh (HRRR) model archives at a 1-hour resolution for the 3km grid-cell containing the study site. Ventilation rate was calculated as the product of windspeed and the mixing height. Except for wind direction, meteorological data were paired to VMS measurements by averaging all the timepoints of a given variable which occurred during a given sampling period. Due to the fact that it is an angular variable, the mean vector wind direction was calculated for each sampling period. In calculating the mean vector wind direction, 0.2 meters per second was used as the cutoff point between “low wind speeds” where windspeed was ignored and the unit direction mean was calculated and “high wind speeds” where wind speed was factored into the direction. Variables obtained from both the anemometer and the HRRR model (temperature, wind speed, and wind direction) were compared. For windspeed and temperature, the relative percent difference was calculated between the average HRRR value and average anemometer value for each sampling period. For wind direction, the absolute circular distance (so that the difference between 359 and 1 is 2, not 358) was computed. The average RPDs for wind speed and temperature were 32±20% and 5.4±4% respectively. The average absolute difference for wind direction was 22±20°. The relationships between VMS concentrations and meteorological variables are shown below in Table S3.

Table S2: Meteorological data from on-site anemometer and NOAA High-Resolution Rapid Refresh model averaged for sampling periods.

Start	End	Temperature (°C) [A]	Wind Speed (m s^{-1}) [A]	Vector Mean Wind Direction (°) [A]	Temperature (°C) [HRRR]	Wind Speed (m s^{-1}) [HRRR]	Vector Mean Wind Direction (°) [HRRR]	Mixing Height (m)	Pressure (mbar) [HRRR]	Dewpoint (°C) [HRRR]	Cloud Cover (%) [HRRR]
7/13/22 8:52	7/13/22 14:09	28	2.7	288	30	2.5	304	1474	1012	14.4	2.5
7/13/22 14:24	7/13/22 20:24	30	4.1	223	31	3.7	234	1479	1011	15.0	7.2
7/13/22 20:52	7/14/22 8:38	25	3.0	88	24	1.8	20	176	1013	18.6	37
7/14/22 14:09	7/14/22 20:24	30	3.8	332	30	2.7	320	1867	1015	14.4	43
7/14/22 20:38	7/15/22 8:38	24	3.2	359	23	2.2	4	196	1018	13.6	7.2
7/15/22 8:38	7/15/22 13:55	25	3.5	89	27	1.9	84	995	1020	13.2	42
7/16/22 8:38	7/17/22 8:38	24	3.1	150	24	2.0	161	370	1017	20.4	76

7/17/22 8:38	7/17/22 12:14	25	1.9	263	26	1.6	195	968	1016	19.2	63
7/17/22 20:52	7/18/22 9:36	26	2.2	138	24	1.3	178	120	1015	21.5	98
7/19/22 14:09	7/19/22 20:24	31	5.2	264	33	3.2	273	1807	1007	17.6	1.9
7/19/22 20:38	7/20/22 8:52	28	3.7	249	27	2.3	267	177	1007	17.6	0.10
7/20/22 14:24	7/20/22 20:09	33	3.7	186	34	3.2	193	1435	1005	19.5	9.5
7/20/22 21:07	7/21/22 8:24	29	4.6	214	27	2.4	209	170	1005	20.6	14
7/21/22 8:52	7/21/22 20:24	30	4.4	186	32	3.2	222	1130	1004	22.1	37
7/21/22 21:07	7/22/22 8:38	29	2.5	269	26	2.1	314	125	1008	17.5	0.0
7/22/22 8:52	7/22/22 13:55	30	3.2	268	33	2.9	287	1564	1011	15.7	15
7/22/22 13:55	7/22/22 20:24	31	3.8	228	33	3.5	252	1014	1011	16.8	30
7/22/22 20:38	7/23/22 8:24	28	2.6	256	26	1.7	316	103	1014	18.5	0.10
7/23/22 8:38	7/23/22 13:26	29	2.3	298	33	3.0	226	1110	1016	15.8	1.3
7/23/22 13:40	7/24/22 12:43	30	3.6	227	30	2.6	225	171	1015	18.0	4.1
7/24/22 12:43	7/24/22 20:38	33	5.0	212	36	3.1	235	2267	1013	16.3	29
7/24/22 20:38	7/25/22 8:24	30	5.8	219	29	2.9	230	466	1012	21.4	49
7/25/22 20:38	7/26/22 8:38	25	3.6	337	24	2.2	331	430	1013	17.9	47
7/26/22 10:48	7/26/22 20:38	25	3.4	272	28	2.9	311	1398	1015	12.5	92
7/26/22 20:52	7/27/22 8:38	24	1.6	334	22	1.3	71	104	1014	14.1	28
7/27/22 16:19	7/27/22 20:24	26	4.0	129	28	3.6	191	679	1012	16.5	100
7/27/22 8:38	7/28/22 12:28	26	2.6	191	26	2.2	192	244	1012	18.5	54
7/27/22 8:52	7/28/22 12:28	26	2.6	192	26	2.2	192	243	1012	18.5	54
7/27/22 12:28	7/28/22 16:04	27	2.8	196	27	2.5	194	246	1011	19.4	53
7/27/22 20:38	7/28/22 8:52	26	2.1	186	24	1.7	198	127	1011	20.8	52
7/28/22 16:19	7/28/22 20:24	30	3.9	164	29	3.5	179	513	1008	22.7	63
7/28/22 8:24	7/29/22 12:14	28	3.3	246	28	2.7	236	371	1010	20.3	43
7/28/22 12:28	7/29/22 16:04	28	3.4	245	29	2.9	245	386	1010	19.7	44
7/28/22 20:24	7/29/22 8:24	27	3.3	263	26	2.2	267	220	1009	19.6	26
7/29/22 16:19	7/30/22 8:38	26	3.6	283	26	2.6	290	340	1013	17.8	67
7/30/22 8:38	7/30/22 14:38	27	5.3	328	29	1.3	315	1514	1016	12.3	0.40
7/30/22 14:52	7/30/22 20:38	29	4.8	328	31	4.0	320	1993	1017	11.6	1.6
7/30/22 20:38	7/31/22 8:24	26	2.9	29	23	1.9	355	135	1020	13.0	0.20
7/31/22 8:24	8/1/22 8:38	26	3.0	201	26	2.6	228	545	1018	17.2	86
8/1/22 8:38	8/1/22 14:09	21	2.6	73	22	2.0	87	541	1015	19.9	99
8/1/22 14:09	8/1/22 20:24	24	2.3	248	23	1.4	230	721	1012	20.2	72
8/1/22 20:24	8/2/22 6:28	24	3.4	225	21	1.3	213	113	1011	19.2	12
8/2/22 6:28	8/2/22 8:24	24	3.7	229	22	1.6	209	263	1010	20.8	15
8/2/22 8:24	8/2/22 12:28	27	3.5	239	28	3.2	242	656	1009	21.5	31
8/2/22 12:43	8/2/22 16:33	31	3.4	259	33	4.0	254	1759	1008	19.6	27
8/2/22 16:33	8/2/22 20:38	32	3.8	263	33	3.6	276	2051	1008	17.4	35
8/2/22 20:38	8/3/22 6:14	27	3.2	340	26	2.0	346	295	1011	17.9	17
8/3/22 6:28	8/3/22 8:24	24	3.0	330	24	2.4	3	371	1015	17.5	0.60

8/3/22 8:38	8/3/22 12:43	26	2.5	305	29	1.6	247	962	1015	16.9	1.5
8/3/22 12:43	8/3/22 16:48	30	2.8	237	33	2.4	246	1699	1014	13.5	1.8
8/3/22 16:48	8/3/22 20:24	30	3.5	163	31	3.6	195	675	1014	16.0	1.6
8/3/22 20:24	8/4/22 8:24	27	2.5	196	25	2.1	NA	221	1015	21.2	9.5

*(A) denotes data obtained from the on-site anemometer. (HRRR) denotes data obtained from the NOAA High-Resolution Rapid Refresh model.

Table S3: R^2 and p-values (listed in parentheses) for linear regressions between VMS concentrations (and natural logs of VMS concentrations) and meteorological variables.

	D3	D4	D5	D6	L5	L7	VMS	lnD3	lnD4	lnD5	lnD6	lnL5	lnL7	lnVMS
Temperature (A)	0.10 (0.03)	0.04 (0.15)	0.02 (0.26)	0.05 (0.1)	0.02 (0.33)	0.08 (0.05)	0.03 (0.19)	0.11 (0.01)	0.1 (0.02)	0.09 (0.03)	0.1 (0.02)	0.07 (0.06)	0.11 (0.02)	0.1 (0.02)
Temperature^{-1} (A)	0.11 (0.02)	0.04 (0.17)	0.02 (0.29)	0.05 (0.12)	0.02 (0.34)	0.08 (0.05)	0.03 (0.21)	0.12 (0.01)	0.1 (0.03)	0.08 (0.04)	0.09 (0.03)	0.07 (0.07)	0.11 (0.02)	0.09 (0.03)
Wind Speed (A)	0.01 (0.46)	0.02 (0.35)	0.01 (0.4)	0.02 (0.36)	0 (0.77)	0.03 (0.26)	0.02 (0.38)	0.06 (0.08)	0.1 (0.02)	0.09 (0.03)	0.08 (0.04)	0.03 (0.26)	0.07 (0.07)	0.09 (0.03)
Mixing Height (HRRR)	<0.01 (0.59)	0.06 (0.07)	0.09 (0.03)	0.08 (0.04)	0.11 (0.03)	0.10 (0.02)	0.08 (0.04)	0.06 (0.07)	0.18 (<0.01)	0.20 (<0.01)	0.18 (<0.01)	0.18 (<0.01)	0.20 (<0.01)	0.19 (<0.01)
Pressure (HRRR)	0.11 (0.02)	0.07 (0.06)	0.03 (0.22)	0.04 (0.18)	0 (0.71)	0.05 (0.12)	0.04 (0.14)	0.09 (0.03)	0.04 (0.18)	0.03 (0.24)	0.02 (0.28)	0.02 (0.31)	0.04 (0.14)	0.03 (0.2)
Cloud Cover (HRRR)	0.07 (0.05)	0.01 (0.55)	0.01 (0.52)	0.02 (0.35)	0 (0.83)	0.06 (0.07)	0.01 (0.45)	0.06 (0.07)	0.03 (0.2)	0.05 (0.11)	0.05 (0.12)	0.05 (0.12)	0.11 (0.02)	0.05 (0.11)
Precipitation (HRRR)	0.05 (0.12)	0.03 (0.25)	0.04 (0.18)	0.06 (0.09)	0.03 (0.25)	0.08 (0.04)	0.04 (0.17)	0.04 (0.13)	0.04 (0.18)	0.05 (0.11)	0.05 (0.11)	0.04 (0.15)	0.08 (0.05)	0.05 (0.11)

*(A) denotes data obtained from the on-site anemometer. (HRRR) denotes data obtained from the NOAA High-Resolution Rapid Refresh model.

Table S4: Median and Mean Log Transformed VMS concentrations for “Day” and “Night”.

Analyte	Median Day Concentration (ng m^{-3})	Median “Night” Concentration (ng m^{-3})	Mean Log Transformed “Day” Concentration (ng m^{-3})	Mean Log Transformed “Night” Concentration (ng m^{-3})	p-value (unpaired t-test between log transformed means)
D3	19	22	1.4	1.4	0.13
D4	74	86	1.8	1.9	<0.01
D5	220	330	2.3	2.5	<0.01
D6	14	16	1.1	1.2	<0.01
L5	2.5	2.8	0.40	0.63	<0.01
L7	1.5	2.8	0.13	0.37	<0.01
Total VMS	330	480	2.5	2.7	<0.01

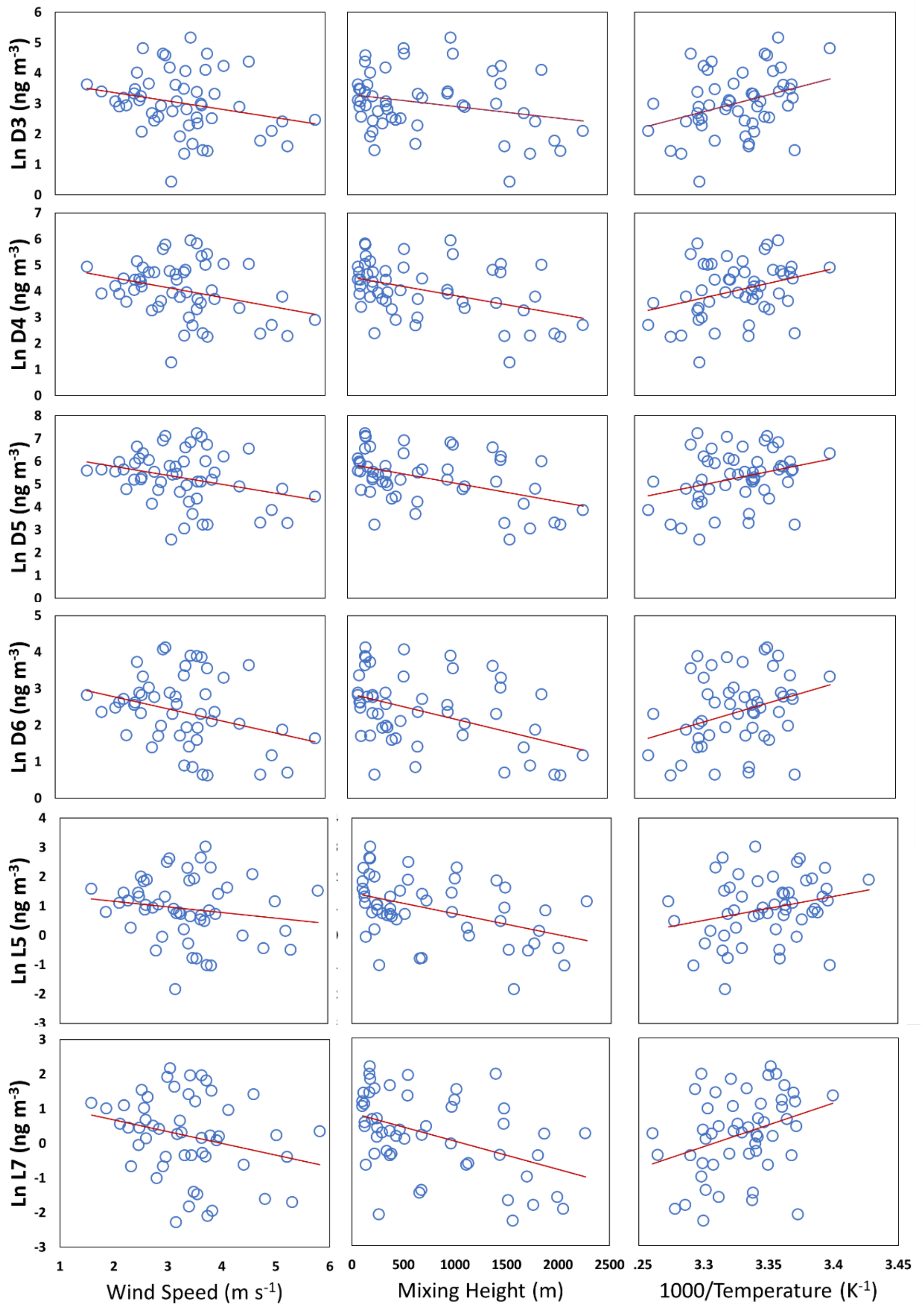

Figure S1: Linear regressions between the natural log of D3 (1), D4 (2), D5 (3), D6 (4), L5 (5), and L7 (6) concentrations and windspeed (A), mixing height (B), and inverse temperature (C).

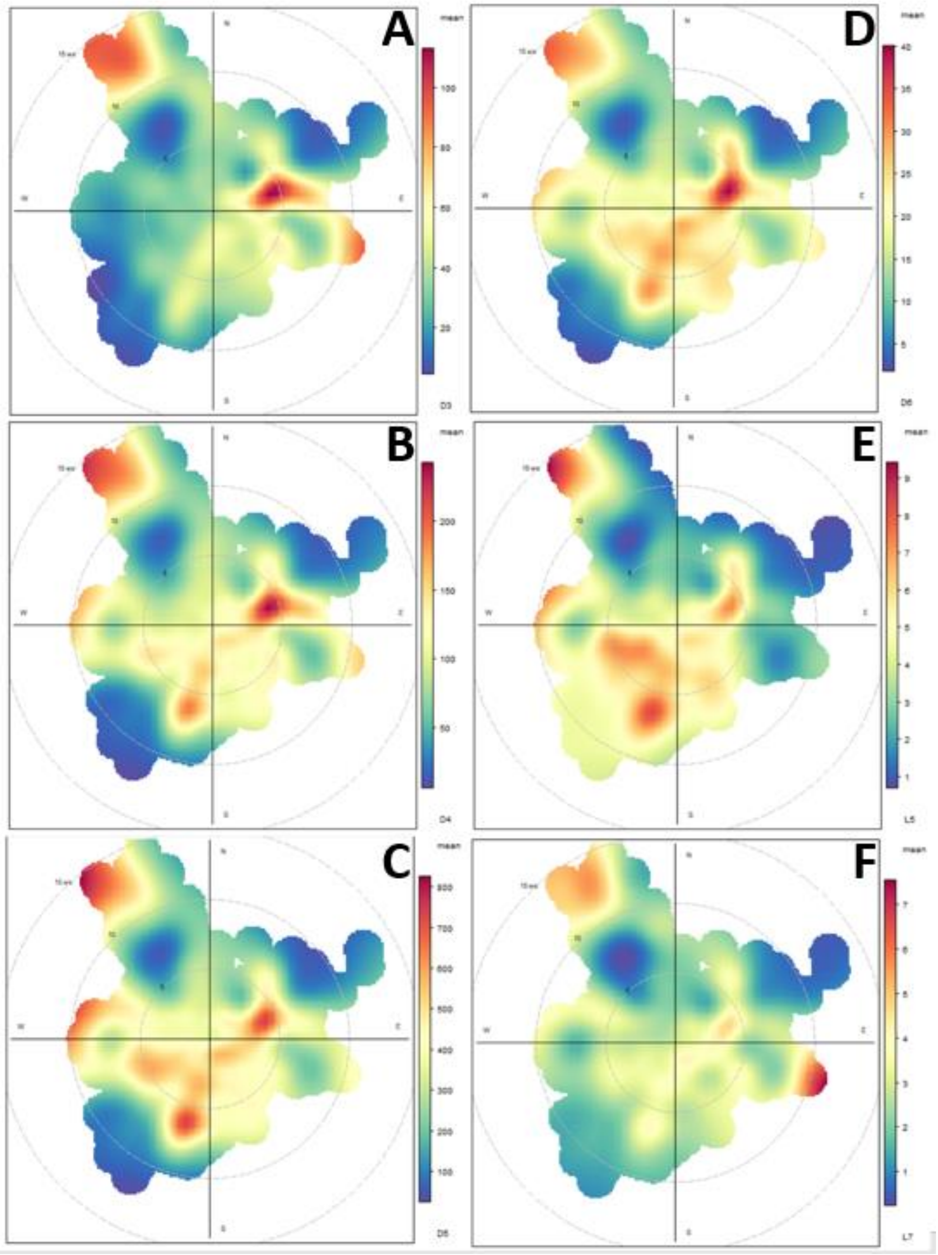

Figure S2: Bivariate polar plots of D3 (A), D4 (B), D5 (C), D6 (D), L5 (E), and L7 (F) concentrations (ng m^{-3}) as a function of windspeed and direction.

Previous Study Locations, Population Density, and Regressions

Table S5 below shows the coordinates, year of sampling, sampling method, and calculated population density for sites that we obtained VMS data from for this study. We selected all published studies we could find that reported the concentrations of at least one VMS congener in outdoor air from non-industrial sites. We intentionally did not include data from one study, Gallego et al. 2017, because despite

occurring primarily in residential areas the authors note that there was considerable industrial activity in the sampling location. While most studies provided the exact coordinates of their study sites, they had to be approximated from maps or descriptions for a small number of studies. For locations that were sampled multiple times (e.g. Toronto) each sampling was treated as a unique site. Population density data for each country was downloaded from WorldPop.org's "Unconstrained individual countries 2000-2020 UN adjusted (1km resolution)" for the specific year that sampling was performed. If sampling was preformed over multiple years, the median sampling year was used. For sampling preformed after 2020, the 2020 dataset for that country was used. For each study site, the 'great-circle-distance' was calculated between the study site location and all 1km grid cells in the matching population density dataset using the 'distVincentySphere' function of the R-package *geosphere* (version 1.5-18), R package. Population density for the site at a selected distance (e.g., 20km) was calculated by averaging the population density values for all grid cells with calculated distance less than or equal to the selected distance.

Table S5: Selected Sites, Sampling Method, and Population Densities

Location	Lat	Lon	Country	Sampling Year	Population Density (capita km^{-2})*	Sampling Method	Citation
Alert (1)	82.5	-63.5	Canada	2009	0	SIP (passive)	Genualdi et al. 2011[1]
Alert (2)	82.5	-63.5	Canada	2014	0	SIP (passive)	Rauert et al. 2018[2]
Alert (3)	82.5	-62.3	Canada	2019	0	STBC Resin (passive)***	Wania et al. 2023[3]
Algonquin	45.6	-78.6	Canada	2019	0	STBC Resin (passive)***	Wania et al. 2023[3]
Andoya	69.14	15.8	Norway	2019	5	STBC Resin (passive)***	Wania et al. 2023[3]
Barcelona**	41.4	2.2	Spain	2014	11269	ENV+ (active)	Companioni-Damas et al. 2014[4]
Barrow (1)	71.32	-156.6	USA	2009	27	SIP (passive)	Genualdi et al. 2011[1]
Barrow (2)	71.32	-156.6	USA	2014	31	SIP (passive)	Rauert et al. 2018[2]
Beijing (1)**	39.9	116.3	China	2017	17094	ENV+ (active)	Jiang et al. 2022[5]
Beijing (2)**	40.2	116.0	China	2017	372	ENV+ (active)	Jiang et al. 2022[5]
Birkenes	58.4	8.3	Norway	2019	12	STBC Resin (passive)***	Wania et al. 2023[3]
Blaebjerg	56.3	8.4	Denmark	2019	21	STBC Resin (passive)***	Wania et al. 2023[3]

Borden	44.3	-79.9	Canada	2019	57	STBC Resin (passive)***	Wania et al. 2023[3]
Boulder	39.9	-105.3	USA	2016	408	PTR-ToF-MS (in-situ)	Coggon et al. 2018[6]
Bratt's Lake (1)	50.2	-104.7	Canada	2009	0	SIP (passive)	Genualdi et al. 2011
Bratt's Lake (2)	50.2	-104.7	Canada	2014	0	SIP (passive)	Rauert et al. 2018[2]
Cape Grim (1)	-40.7	144.7	Australia	2009	1	SIP (passive)	Genualdi et al. 2011[1]
Cape Grim (2)	40.7	144.7	Australia	2014	1	SIP (passive)	Rauert et al. 2018[2]
Cedar Rapids	42.0	-91.7	USA	2011	515	ENV+ (active)	Yucuis et al. 2013[7]
Chicago	41.8	-87.6	USA	2011	4849	ENV+ (active)	Yucuis et al. 2013[7]
Downsview (1)	43.8	-79.5	Canada	2019	3610	STBC Resin (passive)***	Wania et al. 2023[3]
Downsview (2)	43.8	-50.1	Canada	2009	3610	SIP (passive)	Genualdi et al. 2011[1]
Fraserdale (1)	49.9	-81.6	Canada	2019	0	STBC Resin (passive)***	Wania et al. 2023[3]
Fraserdale (2)	49.9	-81.6	Canada	2009	0	SIP (passive)	Genualdi et al. 2011[1]
Fraserdale (3)	49.89	-81.6	Canada	2014	0	SIP (passive)	Rauert et al. 2018[2]
Gortinak	70.2	28.7	Norway	2019	2	STBC Resin (passive)***	Wania et al. 2023[3]
Groton (1)	41.3	-72.1	USA	2009	431	SIP (passive)	Genualdi et al. 2011[1]
Groton (2)	41.3	-72.1	USA	2014	439	SIP (passive)	Rauert et al. 2018[2]
Hilo (1)	19.5	-155.6	USA	2009	0	SIP (passive)	Genualdi et al. 2011[1]
Hilo (2)	19.54	-155.6	USA	2014	0	SIP (passive)	Rauert et al. 2018[2]
Iqaluit	63.7	-68.5	Canada	2019	11	STBC Resin (passive)***	Wania et al. 2023[3]
Karvatn	59.9	8.7	Norway	2019	7	STBC Resin (passive)***	Wania et al. 2023[3]
Kosetice (1)	49.6	15.1	Czech Republic	2009	30	SIP (passive)	Genualdi et al. 2011[1]
Kosetice (2)	49.6	15.1	Czech Republic	2014	30	SIP (passive)	Rauert et al. 2018[2]
Kunming (1)**	24.9	103.0	China	2017	2626	ENV+ (active)	Jiang et al. 2022[5]

Kunming (2)**	24.7	102.8	China	2017	390	ENV+ (active)	Jiang et al. 2022[5]
Kuujiuaq	58.1	-68.5	Canada	2019	9	STBC Resin (passive)***	Wania et al. 2023[3]
Lijian (1)*	26.9	100.2	China	2017	712	ENV+ (active)	Jiang et al. 2023[5]
LiJian (2)*	26.9	100.1	China	2017	512	ENV+ (active)	Jiang et al. 2023[5]
Little Fox Lake (1)	61.4	-135.6	Canada	2009	0	SIP (passive)	Genualdi et al. 2011[1]
Little Fox Lake (2)	61.4	-135.6	Canada	2014	0	SIP (passive)	Rauert et al. 2018[2]
Malin Head (1)	53.4	-7.3	Ireland	2009	30	SIP (passive)	Genualdi et al. 2011[1]
Malin Head (2)	53.4	-7.3	Ireland	2014	29	SIP (passive)	Rauert et al. 2018[2]
Mount Revelstoke	51.1	-118.1	Canada	2014	2	SIP (passive)	Rauert et al. 2018[2]
New York City	40.8	-73.9	USA	2020	10896	ABN (active)	This Study
New York City (2)	40.82	-73.9	USA	2018	10652	PTR-ToF-MS (in-situ)	Coggon et al. 2021[8]
Ny-Alesund (1)	78.9	11.9	Norway	2009	0	SIP (passive)	Genualdi et al. 2011[1]
Ny-Alesund (2)	78.9	11.9	Norway	2014	0	SIP (passive)	Rauert et al. 2018[2]
Paris (1)	48.9	2.4	France	2009	14550	SIP (passive)	Genualdi et al. 2011[1]
Paris (2)	48.8	2.4	France	2020	14092	PTR-ToF-MS (in-situ)	Verma et al. 2023[9]
Paris (3)	48.9	2.4	France	2014	14973	SIP (passive)	Rauert et al. 2018[2]
Point Reyes (1)	38.0	-122.8	USA	2009	14	SIP (passive)	Genualdi et al. 2011[1]
Point Reyes (2)	38.0	-122.8	Australia	2014	14	SIP (passive)	Rauert et al. 2018[2]
Asta	59.3	15.5	Sweden	2009	14	ENV+ (active)	McLachlan et al. 2010[10]
Tystberga	58.8	17.2	Sweden	2011	12	ENV+ (active)	Kierkegaard et al. 2013[11]
Sable Island (1)	43.6	-60.0	Canada	2009	0	SIP (passive)	Genualdi et al. 2011[1]
Sable Island (2)	43.6	-60.0	Canada	2014	0	SIP (passive)	Rauert et al. 2018[2]
Saitama St. 1	35.8	139.8	Japan	2017	8418	PS-2 (active)	Horii et al. 2021[12]
Saitama St. 2	35.9	139.7	Japan	2017	7214	PS-2 (active)	Horii et al. 2021[12]
Saitama St. 3	35.8	139.6	Japan	2017	10093	PS-2 (active)	Horii et al. 2021[12]

Saitama St. 4	35.9	139.5	Japan	2017	3089	PS-2 (active)	Horii et al. 2021[12]
Saitama St. 5	35.9	139.3	Japan	2017	2058	PS-2 (active)	Horii et al. 2021[12]
Saitama St. 6	36.1	139.6	Japan	2017	1685	PS-2 (active)	Horii et al. 2021[12]
Saitama St. 7	36.0	139.7	Japan	2017	1774	PS-2 (active)	Horii et al. 2021[12]
Saitama St. 8	36.2	139.3	Japan	2017	1071	PS-2 (active)	Horii et al. 2021[12]
Saitama St. 9	36.0	139.2	Japan	2017	261	PS-2 (active)	Horii et al. 2021[12]
Storhofdi (1)	63.4	-20.3	Iceland	2009	264	SIP (passive)	Genualdi et al. 2011[1]
Storhofdi (2)	63.4	-20.3	Iceland	2014	221	SIP (passive)	Rauert et al. 2018[2]
Sydney (1)	28.0	-82.2	USA	2009	528	SIP (passive)	Genualdi et al. 2011[1]
Sydney (2)	28.0	-82.2	USA	2014	580	SIP (passive)	Rauert et al. 2018[2]
Toronto (1)	43.8	-79.5	Canada	2010	3428	PUF/XAD-2 (active)	Ahrens et al. 2014[13]
Toronto (2)	43.8	-79.5	Canada	2014	3389	SIP (passive)	Rauert et al. 2018[2]
Toronto (3)	43.7	-79.4	Canada	2019	4509	STBC Resin (passive)***	Wania et al. 2023[3]
Toronto (4)	43.8	-79.5	Canada	2009	3149	PTR-ToF-MS (in-situ)	Coggon et al. 2018[6]
Tudor Hill	32.4	-64.7	Bermuda	2009	467	SIP (passive)	Genualdi et al. 2011[1]
Tudor Hill (1)	32.4	-64.7	Bermuda	2014	459	SIP (passive)	Rauert et al. 2018[2]
Tustervatn	65.8	13.9	Norway	2019	2	STBC Resin (passive)***	Wania et al. 2023[3]
Ucluelet (1)	48.9	-125.5	Canada	2009	21	SIP (passive)	Genualdi et al. 2011[1]
Ucluelet (2)	48.9	-135.5	Canada	2014	21	SIP (passive)	Rauert et al. 2018[2]
Uetliberg	47.4	8.5	Switzerland	2011	1909	ENV+ (active)	Buser et al. 2013[14]
Weilerswist	50.7	6.8	Germany	2019	425	STBC Resin (passive)***	Wania et al. 2023[3]
West Branch, IA	41.7	-91.4	USA	2011	14	ENV+ (active)	Yucuis et al. 2013[7]
Whistler (1)	50.1	-122.9	Canada	2009	22	SIP (passive)	Genualdi et al. 2011[1]
Whistler (2)	50.1	-122.9	Canada	2014	0	SIP (passive)	Rauert et al. 2018[2]

Zeppelin (1)	78.5	11.5	Norway	2011	0	ENV+ (active)	Krogseth et al. 2013[15]
Zeppelin (2)	78.9	11.9	Norway	2019	0	STBC Resin (passive)***	Wania et al. 2023[3]
Zeppelin (3)	79.0	12.0	Norway	2018	0	ABN (active)	Warner et al. 2020[16]
Zhangjiagang (1)	31.9	120.6	China	2017	2564	ENV+ (active)	Jiang et al. 2022[5]
Zhangjiagang (1)	31.9	120.8	China	2017	726	ENV+ (active)	Jiang et al. 2022[5]
Zurich	47.4	8.5	Switzerland	2011	2102	ENV+ (active)	Buser et al. 2013[14]

*Averaged within 10km of the study site
** Coordinates visually estimated from map or site description
*** Styrene-divinylbenzene copolymeric resin

Linear regressions were calculated between the population density values and the D5 concentration for that site (ng m^{-3}) for that site using the “lm” function of the R package *stats*. R^2 values, p-values, and the slope of the regression model are shown in Table S6. for the range of tested distances. The 20km average population density was selected for further analysis of congeners as it had the best fit (R^2=0.6).

Table S6: Log D5 Concentration vs Log Population Density Regression Values

Averaged Population Area (km)	R^2	P-Value	Regression Slope
1	0.56	<0.001	0.29
5	0.57	<0.001	0.30
10	0.59	<0.001	0.31
15	0.54	<0.001	0.30
20	0.49	<0.001	0.29

The same regressions were performed between the 20km average population density and the average concentrations of D3, D4, and D6. The results of these regressions are shown below in Table S7. Linear regressions were not performed for L5 and L7 as data for these congeners was only present at a handful of sites.

Table S7: Log VMS Concentration vs Log Population Density Regression Values

Analyte	R^2	P-Value
D3	0.28	<0.001
D4	0.33	<0.001
D5	0.59	<0.001
D6	0.56	<0.001

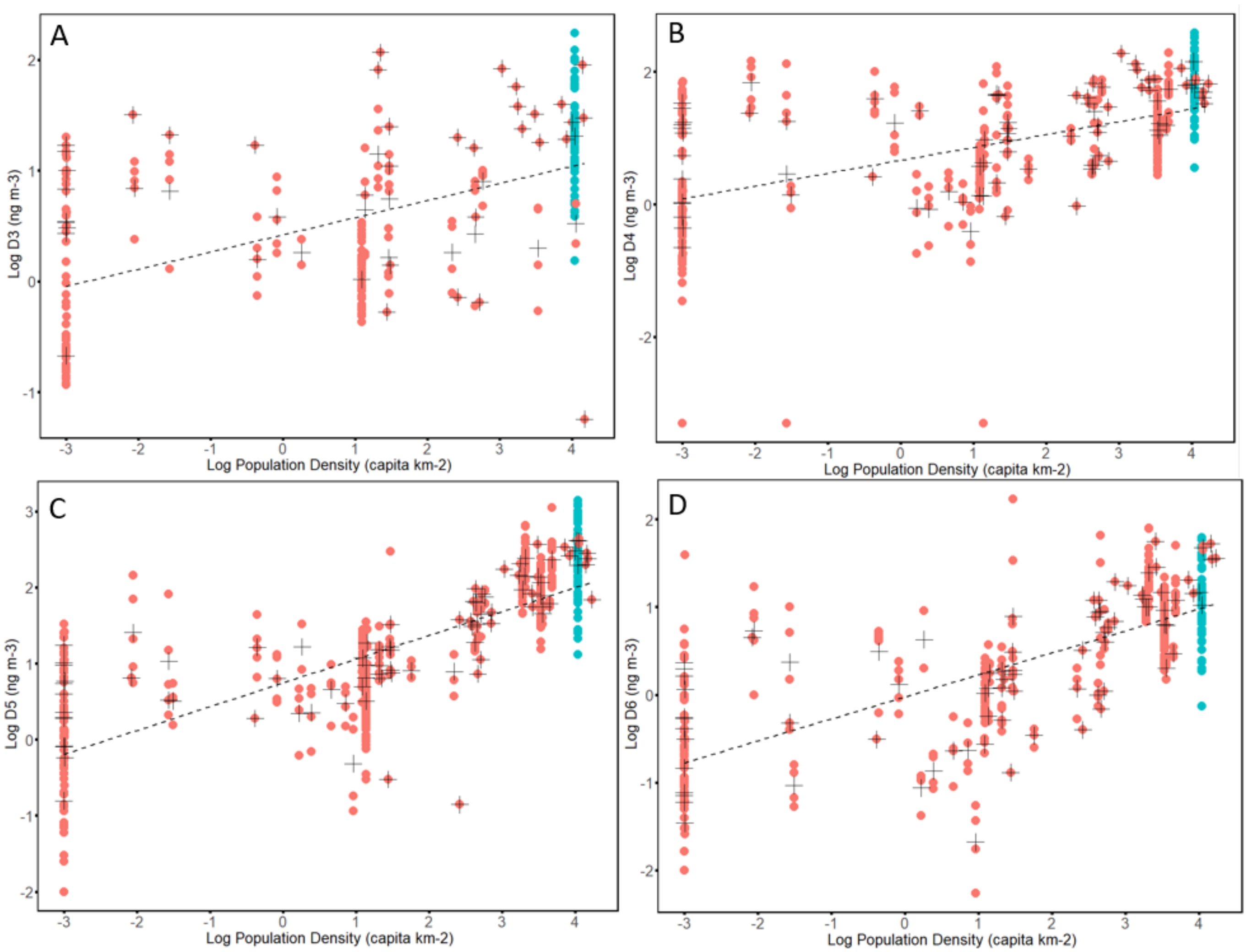

Figure S3: Regressions between Log D3 (A), D4 (B), D4 (C), and D6 (D) and Log Population Density.

Quality Assurance and Control

For each sampling period two cartridges were collected. Both cartridges were extracted and analyzed twice via GC-MS resulting in 4 total mass measurements for each sampling period. The analytical and method variability of the measured samples was assessed by calculating the relative percent difference (RPD) between the calculated masses of each pair of analytical and field duplicates (Equation 1). The average and standard deviation of the analytical and method RPD for each analyte is shown below in Table S8.

[EQ 1]

$$RPD = \frac{ABS(M_1 - M_2)}{0.5*(M_1 + M_2)}$$

Where
M_1 is the mass of replicate 1 (field or analytical).
M_2 is the mass of replicate 2 (field or analytical).

Table S8: Average and standard deviation of relative percent differences (RPD) between analytical and field duplicates collected during the NYC sampling campaign.

	D3	D4	D5	D6	L5	L7
RPD (Analytical)	12±10%	13±11%	13±12%	15±13%	34±25%	19±17%
RPD (Method)	26±25%	29±27%	28±26%	28±24%	46±37%	26±25%

All analyzed cartridges were spiked with known quantities of 13C-D3, 13C-D4, 13C-D5, 13C-D6 prior to extraction. In addition, a solution of pure hexane was spiked with an equal quantity of these surrogate standards and analyzed along with each run. Surrogate recoveries were calculated for each sample per Equation 2 below. Surrogate recoveries for measured samples are shown below in Table S9.

[EQ 2] $$SR\% = \frac{M_S}{M_R} * 100$$

Where
M_s is the mass of surrogate standard measured in the sample.
M_R is the mass of surrogate standard measured in the reference.

Table S9: Surrogate standard recoveries for all analyzed samples.

Sample	13C-D3 Recovery	13C-D4 Recovery	13C-D5 Recovery	13C-D6 Recovery
Average	88%	89%	95%	79%
Standard Deviation	24%	20%	16%	18%
NY-71322-1-1A	78%	78%	83%	73%
NY-71322-1-1B	78%	80%	73%	63%
NY-71322-1-2A	80%	71%	76%	63%
NY-71322-1-2B	92%	92%	85%	84%
NY-71322-2-1A	72%	59%	69%	65%
NY-71322-2-1B	85%	64%	72%	75%
NY-71322-2-2A	103%	72%	72%	71%

NY-71322-2-2B	129%	103%	92%	105%
NY-71322-3-1A	88%	91%	80%	76%
NY-71322-3-1B	86%	74%	69%	79%
NY-71322-3-2A	81%	92%	84%	80%
NY-71322-3-2B	109%	108%	106%	87%
NY-71422-2-1A	162%	125%	124%	92%
NY-71422-2-1B	102%	91%	95%	83%
NY-71422-2-2A	132%	112%	112%	82%
NY-71422-2-2B	108%	106%	104%	85%
NY-71422-3-1A	124%	103%	104%	95%
NY-71422-3-1B	139%	125%	102%	93%
NY-71422-3-2A	87%	71%	76%	73%
NY-71422-3-2B	92%	93%	91%	80%
NY-71522-1-1A	100%	91%	83%	85%
NY-71522-1-1B	81%	94%	91%	67%
NY-71522-1-2A	92%	77%	98%	73%
NY-71522-1-2B	102%	78%	84%	76%
NY-71622-1-1A	102%	61%	78%	63%
NY-71622-1-1B	101%	63%	80%	67%
NY-71622-1-2A	95%	58%	88%	81%
NY-71622-1-2B	114%	74%	102%	91%
NY-71722-1-1A	64%	57%	95%	75%
NY-71722-1-1B	65%	55%	88%	66%
NY-71722-1-2A	72%	65%	99%	75%
NY-71722-1-2B	56%	54%	90%	57%
NY-71722-3-1A	59%	56%	85%	64%
NY-71722-3-1B	52%	57%	85%	71%
NY-71722-3-2A	49%	56%	84%	58%
NY-71722-3-2B	54%	57%	93%	60%
NY-71922-1-1A	59%	78%	88%	67%
NY-71922-1-1B	53%	73%	88%	56%
NY-71922-1-2A	60%	70%	101%	65%
NY-71922-1-2B	55%	75%	96%	65%
NY-71922-2-1A	52%	66%	104%	69%
NY-71922-2-1B	56%	72%	107%	70%
NY-71922-2-2A	63%	87%	112%	70%

NY-71922-2-2B	65%	93%	121%	79%
NY-72022-2-1A	73%	73%	69%	87%
NY-72022-2-1B	113%	102%	116%	132%
NY-72022-2-2A	89%	90%	89%	96%
NY-72022-2-2B	105%	106%	91%	108%
NY-72022-3-1A	90%	84%	84%	90%
NY-72022-3-1B	96%	98%	125%	89%
NY-72022-3-2A	71%	73%	58%	84%
NY-72022-3-2B	102%	103%	94%	100%
NY-72122-1-1A	112%	115%	116%	121%
NY-72122-1-1B	80%	71%	71%	67%
NY-72122-1-2A	95%	96%	97%	103%
NY-72122-1-2B	91%	92%	77%	100%
NY-72122-2-1A	55%	86%	96%	57%
NY-72122-2-1B	57%	90%	105%	64%
NY-72122-2-2A	59%	81%	105%	55%
NY-72122-2-2B	56%	78%	99%	53%
NY-72222-1-2A	59%	82%	90%	62%
NY-72222-1-2B	60%	78%	88%	54%
NY-72222-2-1A	77%	102%	117%	78%
NY-72222-2-1B	68%	96%	110%	72%
NY-72222-2-2A	52%	69%	87%	51%
NY-72222-2-2B	54%	79%	87%	59%
NY-72222-3-1A	62%	79%	99%	63%
NY-72222-3-1B	54%	72%	92%	58%
NY-72222-3-2A	64%	82%	96%	63%
NY-72222-3-2B	60%	82%	93%	60%
NY-72322-1-1A	54%	81%	86%	65%
NY-72322-1-1B	59%	85%	86%	66%
NY-72322-1-2A	67%	96%	106%	62%
NY-72322-1-2B	66%	103%	109%	70%
NY-72322-2-1A	59%	88%	111%	68%
NY-72322-2-1B	62%	88%	124%	72%
NY-72322-2-2A	58%	80%	106%	67%
NY-72322-2-2B	62%	80%	109%	71%
NY-72422-1-1A	48%	71%	80%	46%

NY-72422-1-1B	56%	81%	90%	57%
NY-72422-1-2A	33%	43%	55%	39%
NY-72422-1-2B	35%	49%	56%	33%
NY-72422-2-1A	57%	75%	100%	57%
NY-72422-2-1B	67%	71%	103%	64%
NY-72422-2-2A	54%	65%	88%	60%
NY-72422-2-2B	59%	64%	87%	62%
NY-72522-2-1A	56%	68%	85%	73%
NY-72522-2-1B	56%	59%	79%	67%
NY-72522-2-2A	58%	64%	87%	68%
NY-72522-2-2B	69%	70%	99%	63%
NY-72622-1-1A	93%	59%	88%	81%
NY-72622-1-1B	84%	57%	84%	82%
NY-72622-1-2A	74%	56%	79%	69%
NY-72622-1-2B	72%	55%	83%	68%
NY-72622-2-1A	55%	61%	78%	60%
NY-72622-2-1B	53%	57%	83%	57%
NY-72622-2-2A	55%	59%	83%	55%
NY-72622-2-2B	50%	58%	76%	60%
NY-72722-1-1A	66%	70%	73%	72%
NY-72722-1-1B	57%	59%	80%	65%
NY-72722-1-2A	54%	59%	76%	63%
NY-72722-1-2B	61%	66%	89%	72%
NY-72722-3-1A	88%	93%	82%	69%
NY-72722-3-1B	93%	101%	100%	74%
NY-72722-3-2A	104%	103%	100%	96%
NY-72722-3-2B	99%	69%	94%	105%
NY-72722-4-1A	78%	59%	55%	63%
NY-72722-4-1B	105%	101%	104%	84%
NY-72722-4-2A	90%	102%	84%	70%
NY-72722-4-2B	91%	94%	90%	87%
NY-72822-1-1A	90%	112%	93%	95%
NY-72822-1-1B	92%	113%	86%	71%
NY-72822-1-2A	97%	95%	93%	92%
NY-72822-1-2B	104%	115%	88%	101%
NY-72822-2-1A	103%	107%	83%	85%

NY-72822-2-1B	108%	121%	119%	120%
NY-72822-2-2A	89%	106%	80%	64%
NY-72822-2-2B	69%	80%	76%	66%
NY-72822-3-1A	124%	121%	81%	83%
NY-72822-3-1B	111%	121%	118%	79%
NY-72822-3-2A	106%	126%	80%	76%
NY-72822-3-2B	102%	125%	103%	83%
NY-72822-4-1A	107%	125%	77%	72%
NY-72822-4-1B	105%	113%	102%	90%
NY-72822-4-2A	90%	99%	66%	75%
NY-72822-4-2B	90%	100%	89%	69%
NY-72922-1-1A	97%	101%	73%	71%
NY-72922-1-1B	100%	114%	80%	66%
NY-72922-1-2A	99%	109%	77%	71%
NY-72922-1-2B	98%	111%	81%	93%
NY-72922-2-1A	99%	111%	113%	87%
NY-72922-2-1B	97%	103%	113%	89%
NY-72922-2-2A	108%	105%	104%	92%
NY-72922-2-2B	108%	97%	134%	107%
NY-72922-3-1A	107%	102%	118%	88%
NY-72922-3-1B	115%	119%	120%	103%
NY-72922-3-2A	107%	105%	126%	82%
NY-72922-3-2B	105%	98%	134%	122%
NY-73022-1-1A	102%	99%	114%	87%
NY-73022-1-1B	97%	94%	105%	85%
NY-73022-1-2A	96%	88%	105%	77%
NY-73022-1-2B	95%	93%	85%	113%
NY-73022-2-1A	88%	86%	95%	81%
NY-73022-2-1B	87%	80%	82%	82%
NY-73022-2-2A	103%	95%	91%	75%
NY-73022-2-2B	99%	90%	82%	91%
NY-73022-3-1A	107%	98%	112%	102%
NY-73022-3-1B	101%	104%	88%	86%
NY-73022-3-2A	105%	94%	131%	122%
NY-73022-3-2B	102%	100%	120%	89%
NY-73122-1-1A	96%	88%	93%	85%

NY-73122-1-1B	98%	86%	99%	70%
NY-73122-1-2A	93%	86%	94%	108%
NY-73122-1-2B	91%	99%	93%	74%
NY-80122-1-1A	102%	92%	81%	83%
NY-80122-1-1B	105%	92%	96%	104%
NY-80122-1-2A	104%	90%	97%	75%
NY-80122-1-2B	115%	92%	92%	81%
NY-80122-2-1A	84%	84%	97%	86%
NY-80122-2-1B	87%	82%	90%	97%
NY-80122-2-2A	92%	93%	100%	79%
NY-80122-2-2B	98%	83%	98%	76%
NY-80122-3-1A	94%	88%	102%	96%
NY-80122-3-1B	84%	81%	80%	64%
NY-80122-3-2A	98%	85%	99%	82%
NY-80122-3-2B	105%	104%	115%	78%
NY-80222-1-1A	109%	103%	99%	105%
NY-80222-1-1B	93%	96%	98%	105%
NY-80222-1-2A	95%	90%	99%	73%
NY-80222-1-2B	98%	105%	113%	79%
NY-80222-2-1A	91%	97%	104%	85%
NY-80222-2-1B	64%	54%	70%	50%
NY-80222-2-2A	72%	71%	59%	59%
NY-80222-2-2B	96%	144%	104%	83%
NY-80222-3-1A	75%	86%	86%	64%
NY-80222-3-1B	66%	71%	67%	65%
NY-80222-3-2A	93%	90%	98%	74%
NY-80222-3-2B	100%	102%	107%	103%
NY-80222-4-1A	112%	99%	102%	108%
NY-80222-4-1B	82%	90%	94%	81%
NY-80222-4-2A	121%	120%	105%	122%
NY-80222-4-2B	115%	100%	87%	58%
NY-80222-5-1A	111%	105%	109%	75%
NY-80222-5-1B	111%	99%	110%	101%
NY-80222-5-2A	101%	107%	125%	81%
NY-80222-5-2B	112%	90%	114%	77%
NY-80322-1-1A	115%	115%	124%	109%

NY-80322-1-1B	114%	101%	114%	99%
NY-80322-1-2A	112%	102%	113%	80%
NY-80322-1-2B	122%	113%	124%	72%
NY-80322-2-1A	139%	122%	105%	94%
NY-80322-2-1B	118%	109%	105%	85%
NY-80322-2-2A	118%	113%	90%	128%
NY-80322-2-2B	101%	111%	91%	109%
NY-80322-3-1A	128%	129%	94%	86%
NY-80322-3-1B	117%	124%	111%	101%
NY-80322-3-2A	126%	126%	112%	88%
NY-80322-3-2B	151%	133%	128%	113%
NY-80322-4-1A	109%	117%	105%	115%
NY-80322-4-1B	133%	127%	116%	110%
NY-80322-4-2A	130%	119%	117%	84%
NY-80322-4-2B	113%	101%	114%	98%
NY-80322-5-1A	125%	103%	97%	87%
NY-80322-5-1B	79%	87%	87%	101%
NY-80322-5-2A	87%	73%	78%	59%
NY-80322-5-2B	78%	88%	77%	62%

Field blanks collected throughout the study period and were stored, extracted and analyzed in the same manner as other field samples. LOQs shown below in Table S10 were calculated per Equation 3.

[EQ 3]

$$LOQ = 10^{\left(\overline{\log_{10} m_{FB}} + \frac{\sigma}{\sqrt{n}}\right)}$$

<u>Where</u>

$\overline{log_{10}\, m_{FB}}$ *is the mean of the log transformed field blank masses.*

σ *is the standard deviation of the log transformed field blank masses.*

n is the number of field blanks collected.

Table S10: Field Blank Masses and Limits of Quantification

	D3 (ng)	D4 (ng)	D5 (ng)	D6 (ng)	L5 (ng)	L7 (ng)
LOQ	2.1	4.5	12	0.91	0.34	0.05
FB1	1.7	3.5	9.4	0.66	0.22	0.05
FB2	1.6	2.9	7.9	0.51	0.27	0.04

FB3	1.7	3.9	11	0.98	0.24	0.02
FB4	1.7	3.4	16	1.1	0.23	0.06
FB5	2.4	5.2	7.7	0.56	0.35	0.03
FB6	2.1	4.3	6.9	0.64	0.39	0.05

Breakthrough tests were performed by installing two cartridges in series with each other and extracting and analyzing both in the same manner as field samples (Figure S4). Temperatures and relative humidities during breakthrough testing ranged between 19 and 28 °C and 20-90% respectively. Analyte specific breakthrough was calculated for each sample according to Equation 4. Calculated breakthrough percentages are shown below in Table S11.

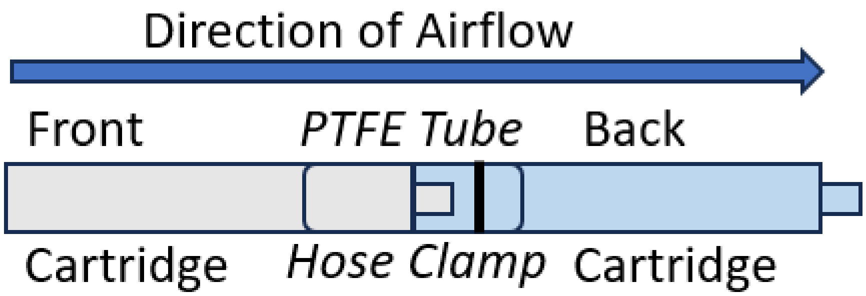

Figure S4: Setup of cartridges for breakthrough tests

[EQ 4] $$BT\% = \frac{M_B}{M_F} * 100$$

__Where__

M_B is the mass of analyte of interest on the back cartridge.

M_F is the mass of surrogate standard measured in the reference.

Table S11: Calculated Breakthrough Percentages

Analyte	BT1	BT2	BT3	BT4	BT5	BT6	Average	Standard Deviation
D3	1%	<1%	<1%	2%	1%	1%	1%	1%
D4	10%	4%	4%	27%	4%	4%	9%	8%
D5	11%	4%	4%	8%	1%	2%	5%	4%
L5	1%	3%	3%	3%	1%	11%	4%	3%
D6	8%	3%	3%	2%	1%	2%	3%	2%
L7	5%	3%	3%	11%	4%	14%	7%	4%

Method Development, Instrument Parameters, and Standard Preperation

Table S12: Chemicals In Use

Chemical	Abbreviation	Purpose	Purity	Manufacturer	Catalog Number
Hexamethylcyclotrisiloxane	D3	Target	98%	Cambridge Isotope Laboratories Inc (CIL). Andover, MA	ULM-9687-S
Octamethylcyclotetrasiloxane	D4	Target	98%	CIL	ULM-9441-MT-S
Decamethylcyclopentasiloxane	D5	Target	97%	CIL	ULM-9442-MT-S
Dodecamethylcyclohexasiloxane	D6	Target	98%,	CIL	CLM-10232-S
Dodecamethylpentasiloxane	L5	Target	97%	Sigma-Aldrich. St. Louis, MO	447269
Hexadecamethylheptasiloxane	L7	Target	97%	AmBeed. Arlington Heights, IL	A207631
Hexamethylcyclotrisiloxane-$^{13}C_6$	^{13}C-D3	Surrogate	98%	CIL	CLM-9542-S
Octamethylcyclotetrasiloxane-$^{13}C_8$	^{13}C-D4	Surrogate	98%	CIL	CLM-9436-MT-S
Decamethylcyclopentasiloxane-$^{13}C_{10}$	^{13}C-D5	Surrogate	98%	CIL	CLM-9437-MT-S
Dodecamethylcyclohexasiloxane-$^{13}C_6$	^{13}C-D6	Surrogate	98%	CIL	CLM-10232-S
2,4,6-tricholorbiphenyl	PCB-30	Internal Standard	98%	CIL	PCB-30
Hexane	Hexane	Solvent	Pesticide Grade	Fisher Chemical Pittsburgh, PA	H300-4
Methylene chloride	DCM	Solvent	Pesticide Grade	Fisher Chemical Pittsburgh, PA	D142-4

A multiple reaction monitoring (MRM) method for the detection of target compounds (D3, D4, D5, D6, L5, L7) surrogate standards (13C-D3, 13C-D4, 13C-D5, 13C-D6) and internal standard (PCB-30) was developed for the Agilent 7000D GC/MS Triple Quad. Mixed standards of the target compounds were prepared in the ~100 ng ml^{-1} concentration range from verified stock solutions and were analyzed using a standard scan method to identify potential precursor ions. The selected precursor ions closely matched both the reported NIST spectrum for each compound and target ions which have been selected in previously developed single ion monitoring (SIM) methods.[1] The prepared standards were then analyzed with a SIM method in order to determine the ideal oven gradient and the exact elution time of each compound. Once these parameters had been determined, a product ion scan method was created using the previously determined oven gradient and precursor ions for each compound. Collision energies for each selected precursor ion were tested between 2 and 20 ev in increments of 2 ev. The

largest ion produced for each compound was selected as the quantitative ion and the second largest was selected as the qualitative ion. A unique collision energy was selected for each transition based on the energy which produced the largest peak area for the given product ion. Based on these tests, a multiple reaction monitoring method was created using the selected product and precursor ions, oven gradient, and collision energies. MRM windows were selected to limit the number of analytes in each window to a maximum of three compounds. The MRM and sample extraction methods were validated with instrument blank, spike recovery, cartridge spike, cartridge blank, and elution volume tests.

The GC (Agilent 7000D Triple Quad with Agilent 7890B GC, Agilent 7693 autosampler, and multimode inlet) was equipped with an Agilent DB5-MS column (fused silica, 30 m × 0.25 mm ID, 0.25 μm film thicknesses) with UHP helium as the carrier gas (constant flow 0.8 mL min^{-1}) and quench gas (0.5 mL min^{-1}) and UHP nitrogen as the collision gas (0.5 mL min^{-1}). The injection volume was 10 μL. The GC inlet operated at the following conditions: initial temperature 45 °C, initial time 0.12 min, ramp 600 °C min^{-1} to inlet temperature 200 °C. The GC oven temperature program was 45 °C for 5 min, 45 to 100 °C at 40 °C min^{-1} and hold for 0.5 min, 100 to 280 °C at 12 °C min^{-1} and hold for 3 min (total run time: 26 min). The MS transfer line temperature was held at 280 °C. The triple quadrupole MS electron ionization source was set to 230 °C and the dwell time to 120 ms.

Precursor ions, product ions, elution times, and collision energies for target analytes are shown below in Table S13. For sample analysis, a calibration standard (90 ng ml^{-1} D3, 90 ng ml^{-1} D4, 90 ng ml^{-1} D5, 90 ng ml^{-1} D6, 94.5 ng ml^{-1} L5, 98.28 ng ml^{-1} L7, 127 ng ml^{-1} 13C-D3, 125 ng ml^{-1} 13C-D4, 123 ng ml^{-1} 13C-D5, 113 ng ml^{-1} 13C-D6, 35 ng ml^{-1} PCB-30), surrogate standard solution (127 ng ml^{-1} 13C-D3, 125 ng ml^{-1} 13C-D4, 123 ng ml^{-1} 13C-D5, 113 ng ml^{-1} 13C-D6) and internal standard solution (500 ng ml^{-1} PCB-30) were prepared in hexane via serial dilution. Each analytical run contained 4 calibration standards and 2 procedural blank samples. In addition, analytical blanks consisting of pure hexane were run between each set of standards and the samples. All samples were analyzed in duplicate. Following analysis, congener peak areas were quantified in Mass Hunter Qualitative Analysis 10.0. The relative response factor (RRF) was calculated for each target compound and the 13C surrogate standards relative to the PCB30 internal standard according to Equation 5. RRFs were calculated for each analyte for four calibration standard injections and then averaged. Analyte masses in samples were then calculated using the average run RRF, the analyte peak areas, and the internal standard mass according to Equation 6.

[EQ 5] $$RRF = \frac{M_{c,C} * A_{is,C}}{M_{is,C} * A_{c,C}}$$

[EQ 6] $$M_{c,S} = \frac{A_{c,S}}{A_{is,S}} * RRF * M_{is,S}$$

Where
$M_{c,C}$=The known mass of the target congener in the calibration standard
$A_{is,C}$ = The measured peak area of the internal standard in the calibration standard
$M_{is,C}$ =The known mass of the internal standard in the calibration standard
$A_{c,C}$=The measured peak area of the target congener in the calibration standard
$M_{c,S}$=The calculated mass of the target congener in the sample
$A_{c,S}$=The measured peak area of the target congener in the sample
$A_{is,S}$ = The measured peak area of the internal standard in the sample
$M_{is,S}$=The known mass of the internal standard in the sample

Table S13: Precursor Ion, Product Ions, Elution Times, and Collision Energies for Target Analytes

		Quantitative Ion			Qualitative Ion		
Analyte	Retention Time (min)	Precursor (M/Z)	Product (M/Z)	Collision Energy (EV)	Precursor (M/Z)	Product (M/Z)	Collision Energy (EV)
D3	6.6	207	191	10	207	73	14
D4	8.5	282	266	12	282	73	10
D5	10.3	355	73	14	355	267	10
D6	12.2	341	73	12	341	325	6
L5	11.3	147	73	12	147	147	6
L7	14.8	221	73	12	221	221	4
13C-D3	6.6	212	195	14	212	76	14
13C-D4	8.5	288	271	8	288	76	14
13C-D5	10.3	364	76	14	364	272	14
13C-D6	12.2	345	74	14	345	329	4
PCB-30	17.1	256	186	20	N/A	N/A	N/A

References

(1) Yucuis, R. A.; Stanier, C. O.; Hornbuckle, K. C. Cyclic siloxanes in air, including identification of high levels in chicago and distinct diurnal variation. *Chemosphere* **2013**, *92* (8), 905-910. DOI: 10.1016/j.chemosphere.2013.02.051.
